# Making a good egg: human oocyte health, aging, and in vitro development

**DOI:** 10.1152/physrev.00032.2022

**Published:** 2023-05-12

**Authors:** Evelyn E. Telfer, Johanne Grosbois, Yvonne L. Odey, Roseanne Rosario, Richard A. Anderson

**Affiliations:** ^1^Institute of Cell Biology, School of Biological Sciences, University of Edinburgh, Edinburgh, United Kingdom; ^2^Centre for Discovery Brain Sciences, Biomedical Sciences, University of Edinburgh, Edinburgh, United Kingdom; ^3^MRC Centre for Reproductive Health, Queens Medical Research Institute, University of Edinburgh, Edinburgh, United Kingdom

**Keywords:** follicle culture, meiosis, oocyte maturation, ovary, reproductive aging, stem cells

## Abstract

Mammalian eggs (oocytes) are formed during fetal life and establish associations with somatic cells to form primordial follicles that create a store of germ cells (the primordial pool). The size of this pool is influenced by key events during the formation of germ cells and by factors that influence the subsequent activation of follicle growth. These regulatory pathways must ensure that the reserve of oocytes within primordial follicles in humans lasts for up to 50 years, yet only approximately 0.1% will ever be ovulated with the rest undergoing degeneration. This review outlines the mechanisms and regulatory pathways that govern the processes of oocyte and follicle formation and later growth, within the ovarian stroma, through to ovulation with particular reference to human oocytes/follicles. In addition, the effects of aging on female reproductive capacity through changes in oocyte number and quality are emphasized, with both the cellular mechanisms and clinical implications discussed. Finally, the details of current developments in culture systems that support all stages of follicle growth to generate mature oocytes in vitro and emerging prospects for making new oocytes from stem cells are outlined.

## Graphical abstract

**Figure:**
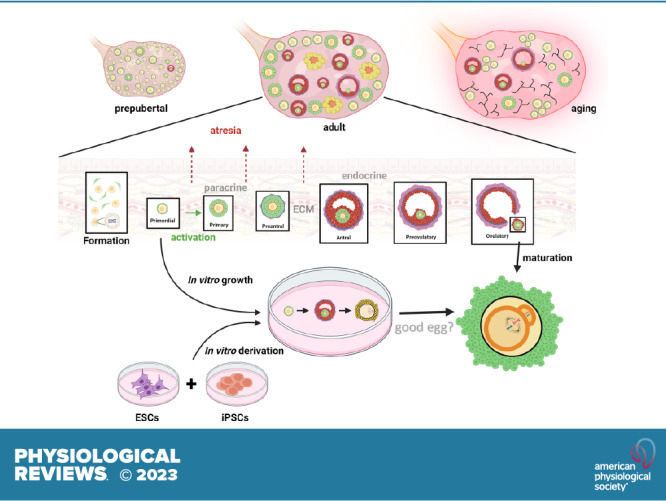

CLINICAL HIGHLIGHTS

1. Follicle formation and the onset of oocyte meiosis occur before birth; thus a female fetus can be directly influenced by exposure to environmental chemicals and maternal factors such as smoking. These can result in transgenerational effects on health and reproductive potential.
2. The regulation of follicle activation and growth remains incompletely understood but is a potential therapeutic target, either to slow it as an approach for protection against damage (e.g., chemotherapy) or to increase it and thus the number of follicles that may be available for stimulation during assisted reproduction.
3. At present, only the very last stages of oocyte maturation can be supported in vitro for clinical use, and even this is infrequently used. Further development of in vitro maturation and progress toward support of follicle and oocyte growth from the earliest stages may lead to dramatic changes in assisted conception in the future.
4. Increasing maternal age at childbirth has major implications for both societies and individuals. Current clinical applications are based on the identification of abnormalities, but a growing understanding of the molecular mechanisms underpinning oocyte meiosis and early embryo development may lead to new therapeutic approaches.

## 1. INTRODUCTION

The mammalian oocyte is the largest cell in the body and develops within the ovarian follicle. The concept of “omne vivum ex ovo”—“all living things come from eggs”—was strongly advocated by William Harvey in 1651 in his classic publication “on the generation of animals” (1), long before Von Baer correctly identified the mammalian oocyte in 1827 (2). A great deal is now known about oocyte development, its structure and regulation within the ovary, and differences between species. The human oocyte has been the subject of much research in the quest to understand the most important single cell in female mammalian reproduction and ultimately to obtain oocytes that could be fertilized in vitro. Experiments were carried out as early as 1944 (3) and following many unsuccessful attempts eventually led to the first birth of a baby in 1978 (4). This early work not only provided a greater understanding of human oocyte development but directly led to the advancement of methodologies that are now in everyday clinical practice and referred to as assisted reproduction techniques (ARTs) (5) or medically assisted reproduction. Ongoing research within this field has demonstrated the potential to develop the most immature oocytes in vitro, while still preserving fertilization and developmental competence, and even the possibility to form new oocytes from stem cells (6, 7). This review details the developmental sequence of the mammalian oocyte within the ovarian follicle from formation to ovulation, outlining information gained from animal models but focusing on what is known about human physiology. The consequences of aging are considered as well as how immature follicles/oocytes can be manipulated in vitro to provide models of human oocyte development and potentially improve fertility treatments for women, concluding with progress on in vitro derivation of oocytes from stem cells.

## 2. ORIGIN OF GERM CELLS

The mammalian ovary has dual functions of producing female germ cells (oocytes) and synthesizing hormones that will regulate the processes of ovulation, fertilization, early embryonic development, and implantation. However, the origin of mammalian germ cells was the subject of fierce scientific debate from the time the mammalian oocyte was correctly identified (2). One side of the debate advanced the argument that germ cells were formed during embryonic development through the proliferation of the so-called germinal epithelium (8). The contrary view proposed that germ cells became segregated from somatic cells before the formation of organ systems and that a continuous germ cell lineage exists through successive generations (9–11). It has been confirmed that germ cells have an extra gonadal origin, and in most mammals, germline cells are established during embryogenesis and segregated from somatic cells before the formation of organ systems.

**FIGURE 1.**
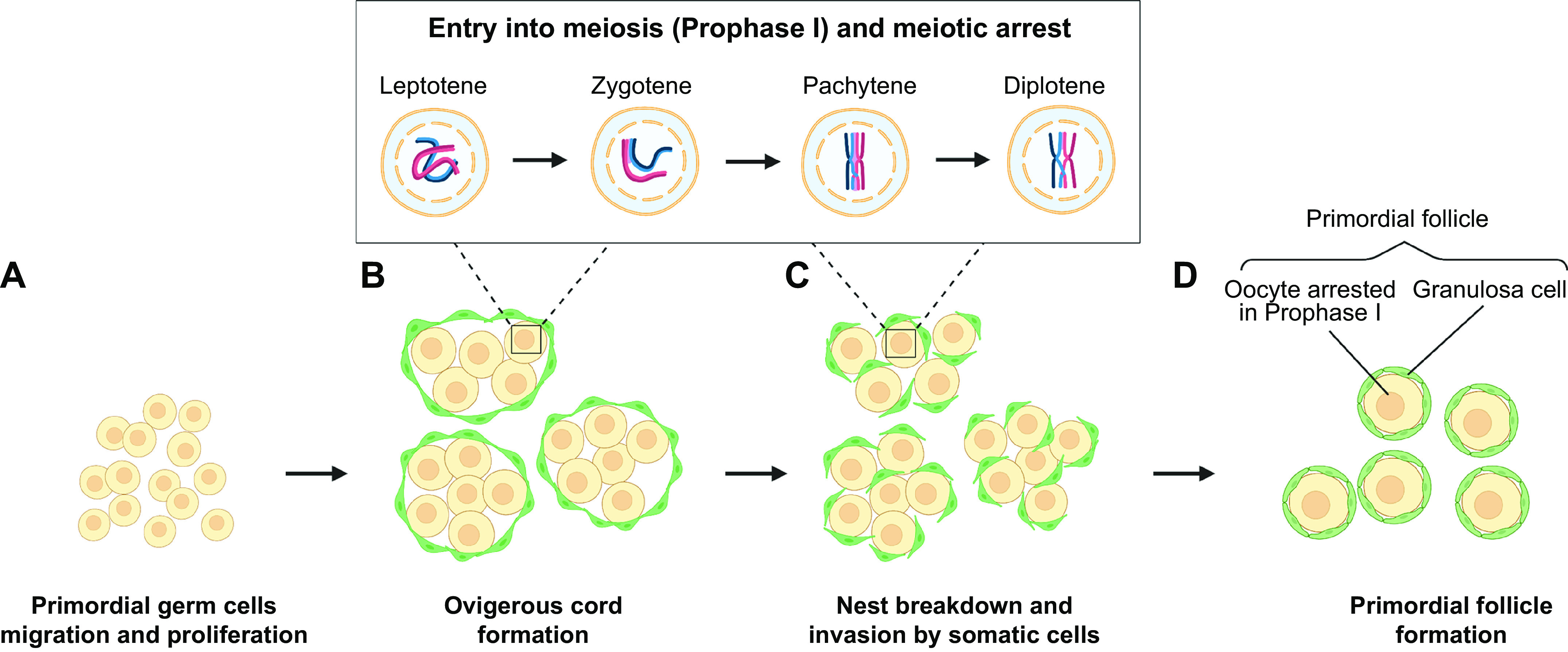
Formation of primordial follicles. Primordial germ cells (PGCs) migrate to the gonadal ridge (*A*) and form nests of oogonia surrounded by somatic cells (*B*) within the presumptive ovary. Oogonia undergo a defined period of proliferation prior to entering meiosis and forming oocytes (*C*). Meiosis progresses to the diplotene stage of prophase I to form oocytes that are found at 16 weeks of gestation in the human fetal ovary. Oocytes establish connections with somatic cells (granulosa cells) to form primordial follicles (*D*). Image created with BioRender.com, with permission.

Primordial germ cells (PGCs) are the progenitors of the germline in all mammals, with the capacity to become either oocytes or spermatogonia depending on the gonadal environment (12, 13). During human embryonic development, PGCs migrate to the genital (gonadal) ridge (14) and within 5–6 weeks postconception can be identified within the indifferent human gonad (15) (FIGURE 1). Sex differentiation is initiated by *week 6* of gestation (16), and it is the somatic environment that determines whether PGCs become sperm or oocytes (17). The absence of sex-determining region Y (SRY) and expression of wingless-related integration site 4 (*WNT4*) and forkhead box transcription factor 2 (*FOXL2*) in the somatic cells induces ovarian development, (reviewed in Refs. 18, 19); thus it is now clear that ovarian development is specifically promoted rather than resulting from the absence of male-specific factors. As PGCs migrate, they express general pluripotency factors (20, 21), which are downregulated after they colonize the developing gonad. This downregulation has been demonstrated experimentally with PGCs losing the ability to give rise to pluripotent cell lines (20, 22). Once primordial germ cells are within the female gonad, they are termed oogonia and will undergo a limited period of mitotic proliferation before entering meiosis and forming oocytes (23) (FIGURE 1). This period of proliferation is noteworthy in that it involves incomplete cytokinesis; thus a syncytium of germ cells is formed (24–26). The formation of nests/cysts in oocyte development is thought to benefit the storage of materials and nutrients required for later development, and this has similarities with the well-described “nurse cell” processes that occur in the *Drosophila* ovary (27). It has been demonstrated in mice that germ cells within the cysts form intercellular bridges that facilitate signaling molecules to synchronize mitosis and meiosis within the cyst (26, 28, 29): similar bridges are present between human germ cells at the same developmental stage (30). The testis expressed 14 (TEX14) protein has been shown to be important in regulating the formation of these intercellular bridges (31) as female mice carrying a mutation for *Tex14* do not form intercellular bridges, but germ cells still form clusters and the females are fertile unlike the males (32).

Recent work has demonstrated that in *Tex14* homozygous mutant fetal ovaries, fewer cysts are formed and germ cells are connected via syncytia or fragmented cell membranes leading to the production of fewer oocytes and many morphologically abnormal oocytes thus demonstrating a direct link between cyst formation, intercellular bridges, and oocyte development (33). Indeed, using novel imaging methods to monitor the development from pluripotency to meiosis in fetal wild-type and *Tex14* mutant mice, it has been shown that cytoplasmic sharing via intercellular bridges coordinates the timing of transition and progression within germline cysts.

## 3. FORMATION OF OOCYTES

**FIGURE 2.**
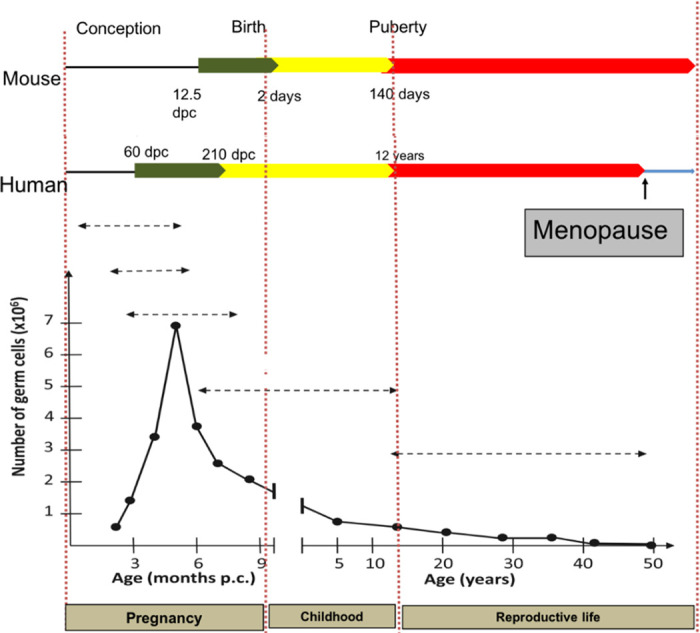
Numbers and timings of oocyte formation and loss. Outline of timings (dpc, days postconception) of follicle formation in mouse and human (green) with graph depicting numbers of germ cells and rate of loss prepubertally (yellow) and until menopause (red). Only 0.1% of follicles will ever be ovulated with the rest degenerating at different stages. Image created with BioRender.com, with permission.

A defining feature of differentiated germ cells is meiosis, the onset of which occurs while they reside in the nest structure and in the human fetal ovary this occurs from 11 to 12 weeks of gestation (25, 34). During this division, oogonia progress to the diplotene stage of prophase I to form oocytes. Oocytes at the diplotene (dictyate) stage of prophase I are found in the human fetal ovary from 16 weeks of gestation, reaching a peak at 19 weeks (35) (FIGURES 1 AND 2). In mice, the initiation of meiosis occurs in a rostrocaudal wave at embryonic day (e)13.5 (36), whereas in the human fetal ovary it is more asynchronous with proliferating oogonia still found at 16 weeks of gestation (35, 37, 38). The organization of the human fetal ovary also differs in that oogonia tend to be found in the outer layers of the ovary, with progressively more mature oocytes and initial primordial follicles found more centrally. Oocyte numbers reach a peak of around 6–7 million at 16–20 weeks in the human ovary (35) (FIGURE 2) and not all oocytes will form follicles, so the numbers are reduced to ∼1 million to 500,000 primordial follicles at term (15, 39). This decline in numbers is regulated by a combination of cell death/survival pathways at each stage of development that will ultimately determine the size of the follicle population (40) and is considered in more detail in sect. 5.

Studies carried out on rodents have shown that retinoic acid (RA) plays a key role in triggering the onset of meiosis (41, 42). RA upregulates the expression of *Stra8* (stimulated by retinoic acid8) a transcriptional activator that enhances the expression of several cell cycle and meiotic prophase 1 genes leading to inhibition of mitotic activity (37, 43); in addition, RA enhances *Rec8* transcription, which encodes a component of the cohesin complex that accumulates during meiotic S phase and is essential for chromosome synapsis and segregation (44). Stra8 has been described as a master regulator as ovaries where *Stra8* has been knocked out do not enter meiosis (45). *STRA8* is expressed in the human ovary and observed between *weeks 9* and *11* (46). Culture systems to support germ cell development in human fetal gonads have provided insight into human germ cell development (47, 48) and have confirmed that RA is necessary for the initiation of meiosis in the human ovary (49).

RA was identified as a prosurvival and proliferation factor for culturing germ cells from fetal mouse ovaries (50), and at e13.5 female germ cells were found to express receptors for RA (50). Early studies on the regulation of the onset of meiosis in female mice identified the requirement for mesonephros (51, 52). It was subsequently found that mesonephric cells produce high levels of RA (53) and that mesonephros is essential for initiating meiosis in mice (54). In mice, RA-synthesizing enzymes are not expressed in the fetal ovary (53), but components required for retinoid synthesis and signaling have been identified in the human fetal gonad (47). These findings support the hypothesis that RA is produced within the human ovary and that it is an ovarian source of RA rather than the mesonephros that is the primary driver of meiotic initiation in the human fetal ovary (47, 48). However, in culture experiments RA is not sufficient to support the progression of meiosis through prophase 1 in all human female germ cells (48) with around half of the germ cells not entering meiosis or degenerating at the zygotene stage (48). This is in contrast with cultured rodent ovaries that readily progress through all stages of meiosis prophase I and form follicles within 10 days (42, 55). These studies suggest nonretinoid extraovarian factors are required for the completion of meiosis 1 in the human fetal ovary (48), although it is possible that suboptimal aspects of in vitro culture might compromise the production of intraovarian support factors. In addition to its essential role in meiosis, evidence from experiments reducing RA production in the fetal ovary supports that it may also be a survival factor for premeiotic germ cells and contribute to the rate of progression of meiosis (56).

During these early stages of meiosis the synaptonemal complex (SC), a protein structure that facilitates pairing of homologous chromosomes, is formed. The SC enables the exchange of genetic material by crossing over and accurate segregation of homologs (57). The proteins that make up the SC, synaptonemal complex protein 3 (SYCP 3) and 1 (SYCP 1), are used as early markers of meiotic onset (58).

The formation of the SC and progression of meiosis is dependent on RNA binding proteins, of which the best characterized is DAZL (deleted in azoospermia-like) and its homologues DAZ (deleted in azoospermia) and BOLL (Bol-like) (reviewed in Refs. 59, 60). *DAZ* is a Y chromosome gene so it is male specific, but *DAZL* and *BOLL* are autosomal. Mice where Dazl has been knocked out are infertile and germ cells do not progress beyond leptotene of meiotic prophase I (61, 62). Germ cells that do not express *Dazl* are unable to express meiotic genes in response to RA (62) and complete synaptonemal complexes fail to form (63). RNA targets in the mouse fetal ovary have been identified and are reviewed in Ref. 59, and this work has been furthered through the identification of human ovarian DAZL RNA targets (64) with roles in regulating chromosome cohesion and DNA recombination, processes fundamental in determining correct meiotic progression and thus oocyte quality. A novel role for Dazl has been indicated in the regulation of germ cell cyst breakdown through *Tex14* (65). Using short interfering RNA knockdown in fetal mouse ovarian cultures, it has been demonstrated that Dazl is required for the timely breakdown of intercellular bridges within germ cell nests and subsequent formation of primordial follicles through translational regulation of Tex14 (65), and this may also be evolutionarily conserved (66).

Although male Boll knockout mice have issues with infertility (67), female mice lacking *Boll* are fertile. This may reflect a period of oogonial development at which *Dazl* is coexpressed and able to compensate for Boll functionality, whereas in the human ovary such coexpression is much more limited (68), allowing for nonredundant functions of BOLL.

During prophase I, homologous recombination and pairing of the homologous chromosomes take place (reviewed in Ref. 69) (FIGURE 1). Recombination facilitates the exchange of genetic material (crossover) by forming double-strand breaks (DSBs) that then require repair. To facilitate this process, the SC forms along each sister chromatid to hold them together and form a synapsis (70). Regulation of DSBs is important as excessive DSBs could compromise genomic integrity but too few could affect the recombination process. Synapsis must be maintained until recombination is complete to ensure alignment and reduction in DSB repair errors (71). Prophase I is divided into four substages (leptotene, zygotene, pachytene, and diplotene) based on defined cytological characteristics (72). Leptotene is defined by the initiation of recombination and chromatin condensing around the forming chromosome axis. The threads of chromatin start to pair homologously during zygotene bringing the chromosome axes closer together. At pachytene, recombination is completed, and in diplotene, chromosomes desynapse in preparation for the first meiotic division (72). The oocyte is then arrested at the diplotene/dictyate stage and will be enclosed within somatic (granulosa) cells to form primordial follicles that will make up the ovarian store of follicles (FIGURE 1).

## 4. FORMATION OF FOLLICLES

As soon as the first oocytes reach the diplotene stage, they are surrounded by somatic cells, the presumptive granulosa cells, and an intact basal lamina encloses the unit to form the primordial follicle. The assembly of primordial follicles leads to the formation of the store of follicles and a number of factors including the balance of death and survival of oocytes during this phase (FIGURE 2) will determine the size of that pool. The intercellular bridges that allow for the passage of cytoplasm and organelles between the cells of the germ cell syncytium break down (73, 74). However, while the formation of bridge structures may not be essential for primordial follicle formation in mice, as follicles form in Tex14-null mice that do not form normal bridges (31), their absence impacts the number and quality of oocytes and ultimately the number of primordial follicles formed (33). High levels of apoptotic cell death take place in mouse oocytes during cyst breakdown. The number of primordial follicles formed is increased if apoptosis is suppressed by deleting genes involved in regulating apoptosis, including caspase 2 or BCL2-associated X protein (Bax) (75); more recently, PUMA (p53 upregulated modulator of apoptosis) has been identified as a key regulator (76). The human fetal ovary expresses myeloid cell leukemia-1 (MCL-1), a member of the antiapoptotic BCL-2 family of proteins, in a gestational age-dependent pattern with higher levels being present during the time of follicle formation (77). Mcl-1 may therefore also be involved in a balance between pro- and antiapoptotic factors regulating cell death during the critical period of interactions with somatic cells during the process of follicle formation. The large family of apoptosis-related factors has a major role in regulating the size of the primordial follicle pool, both during its formation and throughout life (78), as reviewed in Ref. 79, although other cell death pathways are also involved, as discussed below and reviewed in Ref. 80. Indeed, in mice where autophagy has been enhanced, an increased rate of cyst breakdown and follicle formation has been observed (79, 81).

Follicle formation is a key determinant of the size of the pool of primordial follicles and therefore is central to fertility, and while the process has been well described morphologically (82), there is still little understanding of its regulation. The process has been mainly studied in mice (using knockout models and culture systems) with some human data, and several cytokines and growth factors including activin A (83), brain-derived neurotrophic factor (BDNF) (84, 85), tumor necrosis factor-α (TNFα) (86, 87), and kit ligand (KL) (88) have been implicated in its regulation. Despite this, the mechanisms facilitating early interactions and connections between pregranulosa cells and oocytes are still unclear.

The process of primordial follicle assembly in the human ovary is still not well understood (89). The presence of an oocyte is needed for their formation as they fail to form in sterile ovaries (90) or in cases of experimental destruction of oocytes (91) while oocytes that are not enclosed within somatic cells will degenerate. This interdependency between germ and somatic cell components continues throughout follicle development and oocyte maturation and the communication network within the follicle is key to oocyte survival (92). A germ cell-specific transcription factor, factor in germline alpha (FIGLA), plays a key role in regulating these early interactions between the oocyte and somatic cells (93). FIGLA knockout female mice do not form primordial follicles and oocytes are lost shortly after birth (93). *FIGLA* expression rises at the time of primordial follicle formation in the human ovary (94, 95), and mutations have been associated with premature ovarian insufficiency (POI) (96).

Another germ cell-specific transcription factor, Newborn ovary homeobox (Nobox) (97), is also essential at this critical time in determining female fertility (98). Nobox expression is required in mice for cyst breakdown, and when it is absent, the invasion of pregranulosa cells into the cysts is impaired (99) as is oocyte survival and primordial follicle formation (97, 100). Mutations in *NOBOX* have been reported in women with POI with relatively high frequency (101).

These oocyte transcription factors regulate the expression of oocyte-specific factors that play important roles in oocyte development. NOBOX is a regulator of expression of the oocyte-specific growth and survival factor growth differentiation factor 9 (*GDF9*), whose expression also increases immediately before follicle formation in the human ovary (102). These transcription factors and RNA-binding proteins play important roles in controlling the formation of follicles (98, 103).

Recent work links abnormal alternative splicing (AS) of pre-mRNAs with follicle formation. Specifically, serine/arginine-rich splicing factor 1 (SRSF1) is a key posttranscriptional regulator of gene expression in several processes (104). Conditional knockouts (cKO) of *Srsf1* resulted in a reduced number of primordial follicles and complete loss of fertility in female cKO mice. Cyst breakdown and follicle formation were inhibited, and meiosis-related genes were impaired (104). These findings add to our understanding of the mechanisms regulating oocyte meiosis and follicle formation but highlight the complexity of the regulation of these interconnecting processes.

The human ovary can contain ∼500,000 to 1,000,000 oocytes at the time of birth (35). The activation of primordial follicles continues until menopause, when their number is reduced to ∼1,000 (105, 106). This decline is largely due to degeneration following follicular recruitment toward maturation. Several cell death pathways, including apoptosis and autophagy, are involved in regulating this loss at various points during follicle development and are detailed in sect. 5.

## 5. CELL DEATH PATHWAYS DURING FETAL AND PERINATAL LIFE

**FIGURE 3.**
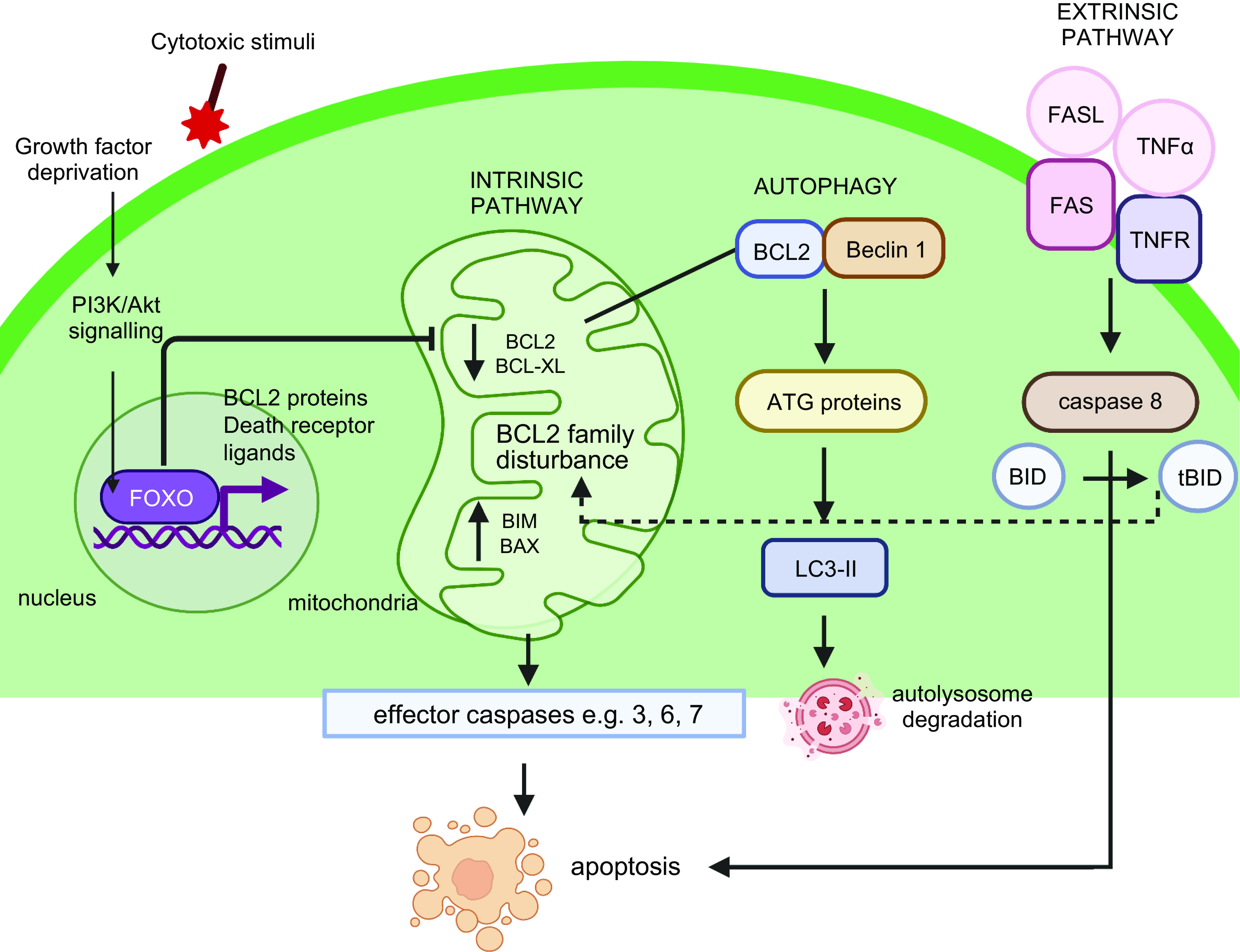
Cell death pathways utilized by oocytes and granulosa cells. The intrinsic apoptotic pathway is elicited through growth factor deprivation or cytotoxic stimuli. Signals are transduced via the phosphoinositide-3-kinase (PI3K) pathway converging on the transcription factor forkhead box O3 (FOXO3) whose targets include BCL2 family proteins and death receptor ligands, causing an imbalance in BCL2 family proteins, activating effector caspases, and leading to apoptosis. Effector caspases are also activated via the extrinsic apoptotic pathway when death receptor ligands binding their corresponding receptors on the cell membrane. Death receptor signaling can also result in BH3 interacting-domain death agonist (BID) cleavage by caspase-8, leading to the generation of active tBID, and cross talk with the intrinsic apoptosis pathway. Finally, BCL2 can interact with Beclin 1 to regulate autophagy, and downstream ATG proteins and LC3-II act to control autophagosome formation. Image created with BioRender.com, with permission.

There are several points during an oocyte’s developmental pathway where tightly coordinated cell death pathways ultimately determine the size of the ovarian reserve and eventually the number of ovulated oocytes (summarized in FIGURE 3 and comprehensively reviewed in Ref. 80). Although this wave of oocyte attrition also occurs in humans (35, 40, 107), the inability to access and manipulate the human fetal and adult ovary for analysis means that most of our knowledge of the pathways underlying oocyte loss has come from studies in the mouse.

The first of these bottlenecks occur during fetal life, with some evidence supporting a loss of oocytes (deemed degenerating by their condensed nuclei with clumps of densely stained chromatin) as early as the last mitotic division (108). Following this, it is unclear whether further oocyte loss occurs steadily during prophase I, as data examining the early stages of meiosis are variable. Little to no germ cell loss has been reported in observations from mouse ovaries at e13.5 to e17.5 (74), while other studies have demonstrated a continuous decline and increased proportion of apoptotic germ cells during the same prophase window (109–112). Nevertheless, the dramatic wave of oocyte attrition that occurs during germ cell nest breakdown and primordial follicle assembly is well documented (74).

Apoptotic mechanisms are fundamental to coordinating germ cell loss at nest breakdown and facilitating primordial follicle formation, with both cleaved poly (ADP-ribose) polymerase 1 (PARP1) and DNA fragmentation being apparent at this time in both mice and humans (74, 109, 113, 114). In addition to DNA fragmentation, apoptosis or programmed cell death is characterized by several other morphological hallmarks including nuclear condensation, membrane blebbing, and cell shrinkage (115). Apoptosis is initiated via intrinsic (mitochondria-regulated) or extrinsic (cell death receptor-related) pathways, which ultimately converge onto the activation of caspase (CASP) family of proteases. Divided into initiator and effector caspases, CASP3 and CASP7 typically act as effectors for the intrinsic pathway, and CASP8 and CASP10 communicate extrinsic pathway signals (78). Although CASP3 is considered the major executioner of apoptosis and is implicated in granulosa cell-driven loss of human and mouse antral follicles, *Casp3*-deficient female mice show no differences in numbers of healthy primordial, primary, or small preantral follicles compared to wild-type littermates on *day 4* postpartum (116), suggesting this caspase is dispensable for perinatal oocyte loss and primordial follicle formation. However, TATA-box binding protein associated factor 4 b (*TAF4b*) null mice exhibit a significant increase in activated *Casp3* immunostaining on *day 1* postpartum compared to control ovaries (117), concomitant with extensive primordial follicle loss, highlighting that different cell death pathways may be elicited under specific physiological conditions. Interestingly, the “nonclassical” effector *CASP2* has been implicated in oocyte death during the perinatal period, as *Casp2*-deficient females had significantly more newly formed primordial follicles when compared with wild-type siblings, suggesting that fetal germ cell attrition was attenuated in the absence of CASP2 (118). Furthermore, oocytes in *Casp2*-null mice exhibited almost complete resistance to the chemotherapy doxorubicin, which caused membrane blebbing and fragmentation in over two-thirds of wild-type cultured oocytes (118). Interestingly, the inactivation of *Casp2* can restore normal oocyte endowment in *Casp11*-null female mice, which would otherwise have significantly reduced numbers of oocyte-containing primordial follicles (119). It is important to note here that given the above mouse models (and many of those discussed throughout this section) are “whole body” knockouts, rather than conditional oocyte or granulosa cell-specific knockouts, it is difficult to ascertain whether the phenotypes observed are due to intrinsic oocyte demise or an oocyte/follicle loss due to lack of granulosa or stromal cell support.

Irrespective of the downstream caspases involved in mediating death signals, it is well established that the key proapoptotic and antiapoptotic members of the BCL2 protein family are clearly required for fetal and perinatal oocyte loss, such members include *BAX* and *PUMA* (proapoptotic) and *BCL2* (antiapoptotic) (76, 120–122). Analysis of BCL2 family proteins in the ovaries of infants and prepubertal girls showed BAX and MCL-1 in oocytes and granulosa cells of all follicle stages, while the BH3 interacting-domain death agonist (BID) was restricted to primordial follicles only (123). BAX is a proapoptotic protein that is highly expressed in degenerating mouse oocytes, while low levels of the protein were observed in many apparently healthy oocytes between e15.5 and birth, when Bax was subsequently downregulated (124). Similar patterns of Bax expression were observed in mouse oocytes in vitro, and it has been hypothesized that Bax-mediated apoptosis in pachytene/diplotene oocytes may act as a meiotic checkpoint to monitor aberrant DNA recombination (124).

Genetic manipulation of *Bax* expression can regulate the size of the ovarian reserve and even extend the reproductive lifespan (125). One study showed that *Bax-*null mice had threefold higher numbers of primordial follicles than their wild-type counterparts (126), while another identified it as a key regulator of oocyte abundance potentially through altering primordial germ cell migration, showing that *Bax* deficiency led to more oocytes and primordial follicles in the embryonic and early postnatal ovary, respectively (127). In addition, targeted disruption of the proapoptotic *Puma* gene caused an increase in germ cell number, but *Puma* was not involved in germ cell nest breakdown but rather a critical regulator of germ cell death during their migratory phase or soon after their arrival in the gonad (76).

BCL2 is considered an oocyte survival factor, and ovaries with C-kit promoter-driven oocyte-specific overexpression of Bcl2 have significantly more primordial follicles than control mice at postnatal day (PND) 12; however, these differences were no longer apparent by PND 30–60 (128). This suggests that additional mechanisms may exist to monitor and remove surplus follicles by adulthood: similar to the removal of excess follicles that are eliminated by PND 19 when treatment of neonatal mouse ovaries with exogenous activin significantly increases primordial follicle number (129). Intriguingly, histological and immunohistochemical examination of *Bcl2* deletion and oocyte-specific *Bcl2* overexpression ovaries at postnatal *days 1*, *4*, and *7* showed no effect on oocyte numbers, nest breakdown, or primordial follicle numbers, despite the steady expression of *Bcl2* in these ovaries (130). Collectively, these data emphasize that many BCL2 proteins may exert their functions during specific developmental windows.

In addition to apoptosis, autophagy plays a key role in culling oocytes during the establishment of the ovarian reserve and follicle loss in postnatal life, with this pathway elicited either specifically by oocytes or granulosa cells (reviewed extensively in Ref. 131). Autophagy is a lysosomal self-dependent degradation process that allows cells to recycle damaged cytosolic components, and despite its primary function as a cell survival pathway, autophagy can lead to cell death in certain circumstances. Oocytes can coexpress autophagy and apoptosis markers (132–134) suggesting an interplay between these pathways in oocyte clearance during development; however, autophagy-mediated cell death that is independent of apoptosis or other regulated cell death pathways can also occur (135–137). For example, antral follicle atresia has been shown to be initiated by massive granulosa cell apoptosis, while preantral follicle atresia is driven primarily by enhanced granulosa cell autophagy (138). In these follicles, the oocyte is eliminated via mechanisms common to apoptosis and autophagy pathways (139–141). Unlike apoptosis, this type of cell death does not result in DNA fragmentation and is independent of *CASP3* and *CASP9* activation (142). Instead, key genes implicated in autophagy include *LC3-II*, *BECN1*, and *ATG7*, as they are determinants of autophagosome formation and elongation (143, 144). Transmission electron microscopy studies of mouse oocytes at PND 0–4 show the frequent presence of autophagosomes in the oocyte cytoplasm and Western blotting of LC3-II expression in protein extracts from ovaries during this period was indicative of ongoing autophagy, with higher levels occurring at the beginning of nest breakdown (145). Genetic manipulation of *Becn1* and *Atg7* in female mice causes a significant reduction in germ cells at PND1 and oocytes at birth, respectively. Furthermore, *Atg7*-deficient mice experience subfertility and a POI-like phenotype later in adult life (146, 147). However, studies that have attempted to induce or inhibit autophagy environmentally or chemically have had mixed results. Autophagy induced by starvation in neonatal mice has produced conflicting results, with evidence of both oocyte loss and impaired primordial follicle formation in one study (145) and augmented primordial follicle formation shortly after birth in others (81, 148). Similarly, observations following treatment with 3-methyladenine to inhibit autophagy at e17 and birth have found reduced oocyte numbers at day 5 of treatment and greater numbers of oocytes in germ cell nests than controls, respectively (149, 150). A possible explanation could be the timing and duration of the autophagic response, with one study showing during the early stages of a 12-h in vitro aging experiment, autophagy increased as an adaptive response to prevent further apoptosis; however, by the late stages, the activation of caspases blocked the autophagic response leading to severe apoptosis (151). Although collectively these findings are difficult to interpret, it remains clear that this cell-death pathway plays a significant role in ovarian reserve formation, and future studies should focus on teasing out its relative contribution to germ cell nest breakdown, oocyte survival, and primordial follicle assembly (134) (FIGURE 3).

## 6. POTENTIAL FOR GERM CELL RENEWAL

Primordial follicles are considered to be nonrenewable and constitute a pool of germ cells that will be utilized throughout life with its size determined by initial formation and the rate of loss of follicles (152). While the oocyte develops within the microenvironment of the follicle, the ovarian stromal environment within which they are embedded is constantly being remodeled, also affecting their fate. Follicles are surrounded by extracellular matrix (ECM) material within the ovarian stroma that provides a supporting scaffold for the developing follicle and a reservoir for paracrine factors. The ECM is essential for supporting the cell-cell interactions and communication needed for follicle formation, development, and migration within the ovary. In addition to ECM, the ovarian environment is composed of stromal cells and a range of cell types including immune cells, nerve cells, and fibroblast cells (153). This creates a heterogeneous environment that regulates a range of cell processes that will contribute to the fate of each follicle. Given the diverse cell types that make up the mammalian ovary (154), there has been an enduring interest in the presence of germline stem cells with the potential to form new follicles.

The capacity of the mammalian ovary to undergo germ cell renewal throughout life was hotly debated in the 1920s (155), and this debate appeared to be settled in the 1950s with the consensus being that postnatal oogenesis did not occur in the mammalian ovary (156). The concept of a fixed population of mammalian oocytes formed before birth (human) or shortly after birth (mouse) became a robust dogma in reproductive biology (156). There are, however, noticeable exceptions such as prosimian primates that have been shown to have a continuous germ line lineage (157–159), and more recently, it has been shown that postnatal oogenesis occurs throughout adulthood in the eusocial mammal the naked mole rat (160).

The possibility of postnatal oogenesis occurring more widely in mammals was given greater consideration following the publication of a study in 2004 that suggested the occurrence of germ cell renewal in adult mice (161). This study was based on counts of follicles and calculation of the rate of cell death and growth with the balance suggesting that maintenance of follicle numbers would require the formation of new follicles postnatally (161). This led to a search to identify the existence of oogonial stem cells (OSCs), also referred to as female germline stem cells (FGSCs) in adult ovaries. The existence of putative germline stem cells in the adult human ovary has also been proposed (162), and putative germline stem cells were subsequently isolated from adult mouse ovaries (163) with the isolation of similar cells from adult human ovaries thereafter (164). Putative germline stem cells have now been isolated from the ovaries of adult mice (163, 164), rats (165), and humans (164, 166–168). Despite the increasing evidence of a cell type with germline potential being present in adult ovaries, the scientific community remains divided with regard to the existence, significance, and derivation of these cells (reviewed in Refs. 169–171).

While some groups have isolated a population of cells with a molecular signature that includes germ and stem cell markers in mice (163, 164), rats (165), and humans (164, 166–168), others have failed to isolate these cells using similar methodologies (172, 173). In human ovarian tissue, this is a rare cell population, comprising ∼0.014% of the total cell population. These cells can stably proliferate in vitro for months and spontaneously generate oocyte-like structures, as determined by morphology and gene expression (164), but freshly isolated cells will also form follicle-like structures in vitro when combined with fetal somatic cells (166).

Isolation of cells with germline potential in the human ovary signifies an important development, but there is a lack of definitive evidence to support these cells playing an active role in replenishing the pool of primordial follicles under normal physiological conditions. Cell depth lineage analysis of oocytes in mice showed increased oocyte depth with age indicating that oocytes ovulated later in life had undergone more mitotic divisions than those ovulated from younger animals suggesting differences in the timing of formation (174, 175). Oocyte depth was further increased in the ovaries of mice following unilateral ovariectomy, suggesting a postnatal renewal of oocytes to compensate for ovarian loss (174). However, other lineage tracing studies in mice could find no evidence to support postnatal oogenesis (28). Evidence suggestive of new follicle formation was found in patients following treatment with a combined chemotherapy treatment, adriamycin, bleomycin, vinblastine, and dacarbazine (ABVD), with increased primordial follicle numbers observed when compared to age-matched control healthy women (176). These results suggest that ABVD treatment may have activated OSCs to form new oocytes/follicles; however, the underlying mechanism for this is yet to be elucidated. Together these results indicate that in women germ cell renewal may occur postnatally under certain physiological or perturbed conditions.

The formation of a pool of follicles before birth with the necessity for many to remain dormant for over 40 years represents a high-risk evolutionary strategy, so there is a need for mechanisms to ensure the fidelity of oocytes during the dormant period. Germ cells are vulnerable to oxidative stress due to damaging reactive oxygen species (ROS), which are by-products of mitochondrial activity (177). Recent studies have demonstrated that human oocytes have developed a highly conserved mechanism, also present in *Xenopus*, to avoid being exposed to damaging ROS while in the dormant phase (178). Dormant oocytes within primordial follicles have a mitochondrial adaptation that results in low complex 1 activity and thus low levels of ROS, which changes when oocytes are activated to grow (178).

## 7. PRIMORDIAL FOLLICLE RECRUITMENT INTO THE GROWING POOL

Primordial follicles are maintained in a state of growth arrest, characterized by a low transcriptional and translational activity, essential for preserving chromosomal stability and prolonged reproductive life. Primordial follicle activation (PFA) is the process by which primordial follicles are selected into the growing follicle pool and is characterized by the differentiation of the flattened granulosa cells into mitotic cuboidal granulosa cells before oocyte growth (179–181).

**FIGURE 4.**
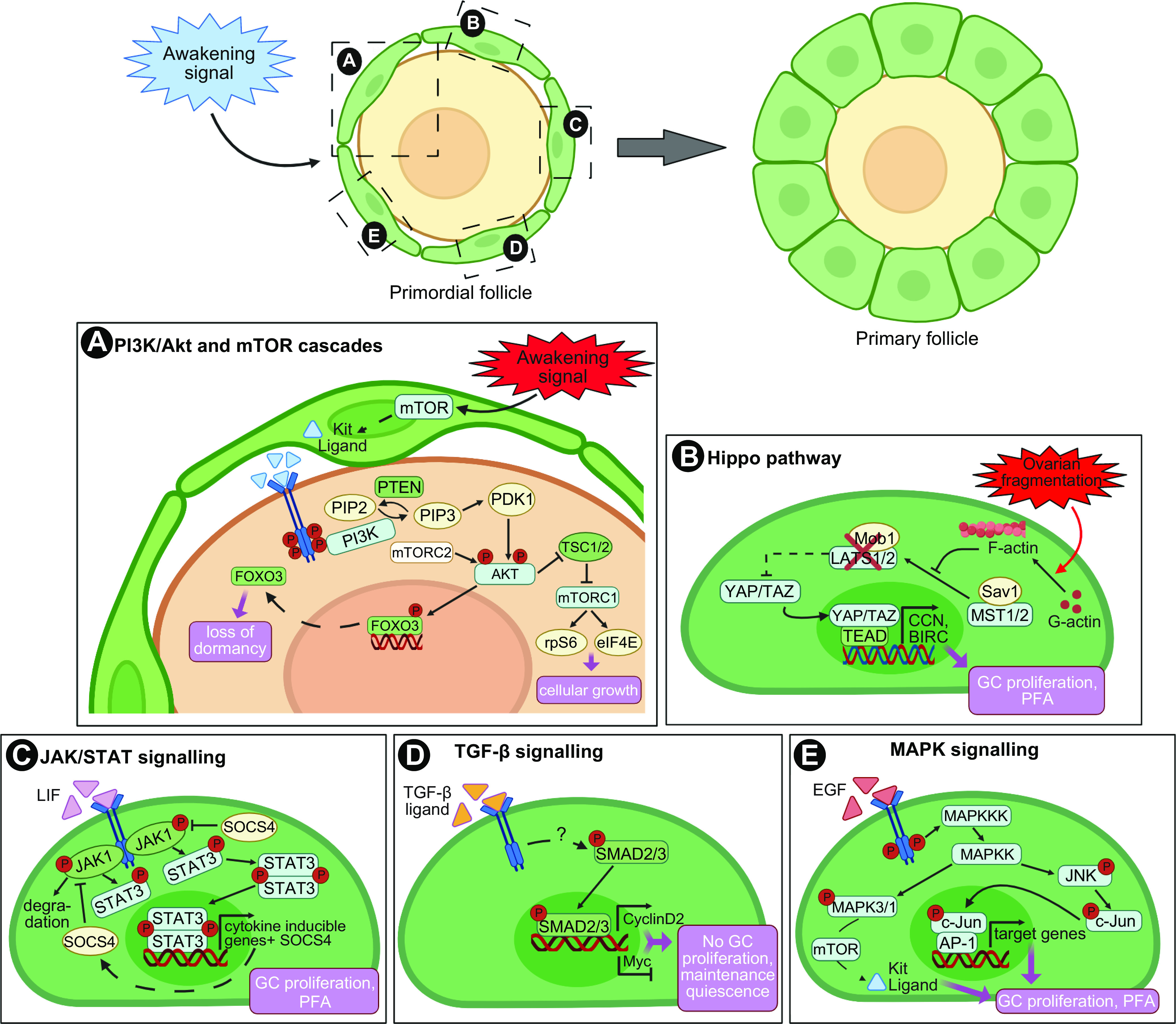
Intrafollicular signaling pathways regulating primordial follicle quiescence and entry into growth. *A*: granulosa cell (GC) induction of mammalian target of rapamycin (mTOR) leads to the secretion of kit ligand that binds its c-KIT receptor on oocytes, triggering the phosphoinositide-3-kinase (PI3K) cascade. Phosphorylation of AKT triggers nuclear export and suppression of forkhead box O3 (FOXO3) transcription factor activity to promote follicle activation, and induces the activation of the downstream mTOR pathway components to direct cell growth. *B*: Hippo dysregulation by ovarian fragmentation triggers a switch in the G-actin/F-actin ratio, resulting in the inhibition of LATS1/2 activity and YAP1 dephosphorylation and translocation into the nucleus. YAP/TAZ interaction with TEAD transcription factors promotes the expression of target genes involved in granulosa cell proliferation and primordial follicle activation (PFA). *C*: activation of the JAK/STAT pathway leads to STAT3 phosphorylation and formation of dimers that translocate to the nucleus, bind to DNA, and regulate transcription of genes involved in GC proliferation and primordial follicle activation. The JAK/STAT activity is negatively regulated by SOCS4. *D*: GCs from quiescent follicles express the transcription factor SMAD3, which promotes expression of cyclin D2 and represses Myc. Cyclin D2 is bound by the inhibitory factor P27 preventing cell cycle progression while repression of Myc maintains growth arrest. *E*: activation of the MAPK signaling triggers the phosphorylation of MAPK3/1, which participates in mTOR pathway activation, and JNK, which controls the activity of the proto-oncogene c-Jun and downstream transcription factor AP-1, both promoting GC proliferation and follicle entry into growth. Granulosa cells in green, oocyte in yellow. Image created with BioRender.com, with permission.

The balance between primordial follicle dormancy and activation is complex, involving numerous molecular, cellular, and biochemical events, whose interactions continue to be characterized (182) (FIGURE 4). Given that primordial follicles lack functional gonadotropin receptors (183) and have limited access to the blood supply, PFA is believed to be controlled in a gonadotropin-independent manner, relying on paracrine signaling within the follicle and across the local environment. Extensive research using genetically modified mice, in vitro culture experiments, and transcriptomic analysis has enabled considerable progress toward understanding the intricate signaling network that regulates follicle activation. Some of these pathways are outlined here and summarized in FIGURE 4 and are discussed further in sect. 7.3. Additionally, an alternate hypothesis has recently been proposed for follicle activation and depletion based on the activity of the integrated stress response (ISR) (184). The ISR is a common pathway within cells and is active in states of cell stress, e.g., DNA damage (185), preventing cell replication. Conversely, when these factors are resolved, cell growth occurs. Granulosa cells treated with TNF-α show immediate increases in activation of ISR-related gene products including cell cycle checkpoints, with the authors hypothesizing that resolution of this effect and thus a low ISR state result in cell proliferation and by extension follicle growth activation (184). Data supporting this remain limited, particularly in relation to the oocyte, but it might provide a common pathway integrating various intracellular damage sensing pathways and intercellular signaling.

### 7.1. Phosphoinositide-3-Kinase/Protein Kinase B and Mammalian Target of Rapamycin Cascades

The phosphoinositide-3-kinase (PI3K)/protein kinase B (AKT) and mammalian target of rapamycin (mTOR) signaling pathways are crucial for fundamental cellular processes, from cell growth and proliferation, survival, and migration to metabolism (186). In mammals, the PI3K cascade is initiated by insulin and growth factors such as kit ligand (KL), insulin-like growth factor-1 (IGF-1), or epidermal growth factor (EGF). Upon ligand binding, activated PI3K catalyzes the phosphorylation of phosphatidylinositol-4,5-bisphosphate (PIP_2_) into phosphatidylinositol-3,4,5-triphosphate (PIP_3_). This reaction is reversed by the phosphatase and tensin homolog deleted on chromosome 10 (PTEN), which converts PIP_3_ into PIP_2_. PIP_3_ recruits phosphoinositide dependent-kinase 1 (PDK1) and Akt to the membrane, where PDK1 phosphorylates AKT. Further phosphorylation of AKT by the mammalian target of rapamycin complex 2 (mTORC2) leads to the full activation of the protein, which mediates the regulation of protein synthesis, cell survival, and cell cycle entry through targeting forkhead box O3 (FOXO3), Bad, tuberous sclerosis complexes 1/2 (TSC1/2), and Cdk inhibitor p27 (187). Notably, AKT translocates into the nucleus of the oocyte and phosphorylates FOXO3, resulting in its export into the cytoplasm and abolishing its profollicular dormancy activity. Another target of AKT is mTOR, a serine/threonine kinase. Phosphorylation of TSC2 by Akt destabilizes the TSC1/2 complex, releasing its inhibitory effect on mTOR and upregulating mTOR activity. Active mTORC1 phosphorylates and activates its downstream effectors 70 S6 kinase 1 (S6K1), ribosomal protein S6 (rpS6), and eukaryotic translation initiation factor 4E (4E-BP1), promoting cell growth through protein translation and ribosomal biogenesis (188). AKT-induced phosphorylation of p27, a major suppressor of cell cycle progression, triggers its shuttling from the nucleus to the cytoplasm and opposes G_1_ arrest (189) (FIGURE 4*A*).

The physiological significance of the PI3K/Akt and mTOR pathways in ovarian follicles was confirmed using genetically modified mouse models. Loss of function of inhibitors of PFA, such as Pten, Tsc1, Foxo3a, and p27, leads to the overactivation of the entire pool of primordial follicles and early depletion of the ovarian reserve leading to premature ovarian failure in mice (190–196). Conversely, constitutively active Foxo3 in oocytes retards oocyte growth and follicular development, causing anovulation and infertility (197), while deletions of Pdk1 and rpS6 trigger follicular loss via accelerated atresia (198). In humans, analysis of the transcriptomic profiles of oocytes from early stage follicles revealed an upregulation of both PI3K/AKT and mTOR pathways during the primordial to primary follicle transition, while PTEN signaling decreases (199, 200). Moreover, higher concentrations of mRNA for AKT1, TSC2, mTOR, and S6K are found in the peripheral blood of women with POI compared with controls (201), and FOXO3 mutations have been reported in some women with POI (202). These data suggest a conserved role for the PI3K/AKT and mTOR cascades in regulating PFA in mice and larger mammals, including in humans. Kit ligand-KIT receptor tyrosine kinase (c-Kit) signaling was later identified as the pivotal link between the mTORC1-KL cascade in granulosa cells and c-Kit/PI3K signaling in oocytes for governing PFA (203). The awakening signal is first perceived and processed by the granulosa cells through the activation of the mTOR cascade, leading to the secretion of KL. KL then binds to its receptor, c-KIT, at the oocyte surface, which activates the PI3K/AKT pathway in the oocyte and eventually ensures coordinated oocyte growth with granulosa cells becoming cuboidal and undergoing proliferation (203).

Taking advantage of the increasing characterization of the mechanisms underlying the PFA process, many drugs targeting either individual or multiple components of the PI3K/AKT and mTOR pathways have been developed for fertility preservation purposes. Chemical activation of mTOR using MHY1485 (204, 205) or propranolol and phosphatidic acid (206, 207) induces PFA in both mouse and human ovaries. Similarly, short-term exposure of ovaries in vitro to bisperoxovanadium compounds [bpV(pic) or bpV(HOpic)], which are PTEN inhibitors, triggers PFA in mice (208, 209), sheep (210), pig (211), bovine (212), and human models (213–215). Conversely, maintaining follicle quiescence is an interesting strategy to limit the potential gonadotoxicity of cancer treatments on the follicular pool. In vitro treatment of rat ovarian granulosa cells with mTOR inhibitors, such as rapamycin, everolimus, or temsirolimus, reduces cell proliferation without affecting cell survival (216). Rapamycin treatment prevents PFA and preserves the ovarian reserve both in vivo and in vitro in mice (203, 217–222) and rats (223). Rapamycin and everolimus have also been reported to protect the ovarian reserve against chemotherapy-induced early follicular exhaustion in mice by maintaining primordial follicles in a dormant state (224–227).

### 7.2. Hippo Signaling Pathway

The Hippo pathway is highly conserved in mammals and regulates organ size via control of cell proliferation, apoptosis, and stem cell self-renewal (228, 229). It consists of a core kinase cascade of negative regulators of growth and is regulated by the cytoskeleton and the surrounding structural environment, responding to changes in both intracellular and extracellular cues such as cell-cell contact, cell polarity, energy stress, and some G protein-coupled receptor ligands (230–233). The central components of the Hippo pathway are the kinases mammalian Ste-20 like kinase 1/2 (MST1/2) and large tumor suppressor homolog 1/2 (LATS1/2), and their regulatory proteins, Salvador (SAV1), and MOB kinase activator 1 (Mob1), respectively. Under basal conditions, the MST/SAV1 complex phosphorylates and activates LATS1/2-Mob1, which in turn phosphorylates and inactivates Hippo’s downstream effectors yes-associated protein 1 (YAP1) and transcriptional coactivator PDZ-binding motif (TAZ) via their sequestration and proteolytic degradation in the cytoplasm. Upon Hippo disruption, unphosphorylated YAP1 and TAZ translocate into the nucleus and bind to TEAD transcription factors (TEAD1–4), promoting the expression of target genes such as CCN growth factor, baculoviral IAP repeat containing (*BIRC*), and the cell cycle regulator protein c-Myc (234, 235) (FIGURE 4*B*).

The role of the Hippo signaling pathway in the ovary and during follicle activation and growth is being increasingly documented. Gene expression studies and immunostaining approaches have demonstrated the expression of Hippo components in mouse, bovine, and human follicles (215, 236–241). Mouse model experiments including granulosa cell-specific deletions for *Lats1*, *Lats2*, *Yap1*, and *Ccn2* have been associated with enlarged ovaries, subfertility, and impaired follicle development (242–245), and an in vitro study reported that Yap1 knockdown attenuates follicle growth while its overexpression promotes PFA (238). However, oocyte-specific deletion of *Yap1* in mice has no impact on primordial follicle formation, activation, and folliculogenesis (246), suggesting that Hippo signaling directs follicular growth in ovarian somatic cells rather than germ cells. This was further confirmed by computational data that identified the YAP/TAZ signaling pathway as active in somatic cells during mouse primordial to primary transition in vivo (247) and in vitro studies demonstrating the nuclear shift of YAP within the granulosa cells of human primordial follicles during PFA (215). In addition, aberrant Hippo signaling and genetic variants of YAP1 have been correlated with enhanced susceptibility for polycystic ovary syndrome (PCOS), characterized by enlarged ovaries (248, 249), while gene copy variations for *BIRC1* have been reported in women with POI (250).

Modulation of the local mechanical forces by ovarian fragmentation has been associated with increased actin polymerization and disrupted Hippo pathway, leading to a shift of YAP from the cytoplasm to the nucleus and transcription of target genes promoting follicular growth (215, 236, 237, 239). Similarly, actin polymerization drugs have been shown to effectively disrupt the Hippo pathway, although associated with variable success in initiating PFA (251, 252). Likewise, ovarian wedge resection and drilling have been used clinically to relieve inhibition of follicle growth and induce ovulation in PCOS patients, with promising results (253). The potential benefit of physically disruptive procedures has clinical relevance for boosting the activation of the follicular pool to rescue the residual follicles present in the ovaries of women with POI and diminished ovarian reserve (254, 255).

### 7.3. In Vitro Activation: Clinical Application

In recent years, in vitro activation (IVA) has emerged as a new therapy that initially relied on the combination of Hippo disruption and PI3K/Akt upregulation to activate residual primordial/small follicles in women who had a low ovarian reserve. The procedure involved the surgical removal of cortical strips from the ovary, their fragmentation, and short-term culture with Akt stimulators and then their transplantation back into the patient (208, 239). This technique successfully promoted follicle growth, allowed the isolation of mature eggs, and led to the delivery of healthy babies from some POI patients, even after cryopreservation of ovarian cortical tissue (239, 256–258). Yet, several in vitro studies have reported bpV-induced follicular damage, including low survival of growing follicles, morphological abnormalities, and DNA repair defects (212, 213, 215, 259). As such, IVA has since been refined into a shortened, drug-free procedure, sufficient to promote follicle growth and maturation. Pregnancies in women with POI and poor ovarian response have been reported (260–263), but the safety, efficiency, and convenience of IVA remain uncertain (264–267).

### 7.4 Other Regulators of PFA

In addition to the pathways described above, additional pathways have been described as potential regulators of PFA (FIGURE 4, *C*–*E*). A recent upstream regulator analysis during mouse PFA identified several potential upstream molecules that either positively or negatively regulate downstream target gene expression in the transitioning granulosa cells, including members of the WNT, mitogen-activated protein kinase (MAPK), and transforming growth factor-β (TGF-β) signaling (268). A transcriptomic study in human granulosa cells reported similar data, with the downregulation of the TGF-β, Janus kinase/signal transducer and activator of transcription (JAK/STAT), and MAPK pathways in granulosa cells during the primordial-to-primary follicle transition, while the Wnt family signaling was enriched (269). TGF-β signaling plays an important role in the maintenance of the primordial follicular pool and regulation via growing follicles.

Anti-Müllerian hormone (AMH) is produced by granulosa cells, and its synthesis, initiated in primary follicles, reaches its highest levels in preantral and small antral follicles and then sharply declines in large follicles (270, 271). There is a general consensus that AMH regulates the rate at which the ovarian reserve is depleted by inhibiting PFA in mammals. AMH knockout mice exhibit a reduced number of primordial follicles and an increased number of preantral and small antral follicles (272), while AMH treatment inhibits PFA in explant cultures of mouse, rat, and goat ovarian tissue (273–275). In humans, conflicting results regarding the suppressive effect of AMH on primordial follicles have been reported using cultured human ovarian cortex (276, 277). Nevertheless, the progressive decline of circulating AMH levels with increasing age occurs concomitantly with an accelerated follicle loss (278–280), and mutations in the AMH and AMHR2 genes are associated with POI or decreased age at menopause (281, 282).

Density-dependent interfollicular regulation of PFA via local diffusing inhibitory factors has been postulated (283), and follicle-follicle interactions play an important role in these processes (284). Candidate inhibitory signals include members of the TGF-β superfamily, such as AMH as described above and TGF-β1 (283). Culture of mouse and rat neonatal ovaries with TGF-β1 reduces the population of activated follicles, while inhibition of type I TGF-β receptors accelerates oocyte growth and granulosa cell proliferation (285, 286). By mediating cell cycle arrest in granulosa cells, the TGF-β signaling mediators SMAD2/3 drive cell proliferation in the granulosa cells of growing follicles (179, 287). Likewise, activation of the JAK/STAT pathway is linked with the morphological changes associated with granulosa cell activation, and treatment of mouse ovaries with leukemia inhibitory factor (LIF) or the JAK inhibitor Ruxolitinib upregulates STAT3 and SOCS4 protein expression, increases apoptosis, and accelerates PFA (288, 289). Further evidence also suggests the involvement of MAPK signaling during primordial follicle recruitment. Beyond the fact that extracellular-signal-regulated kinase 1/2 (ERK 1/2) participates in mTORC1 pathway activation to trigger PFA (290, 291), pharmacological inhibition of members of the MAPK family has also been shown to block the onset of folliculogenesis in cultured mouse, rat, and sheep ovaries (290, 292). This effect is likely through its interaction with the mTORC1-KITL signaling pathway in pregranulosa cells and the KIT-PI3K signaling in oocytes (290, 292). Recent studies suggest an involvement of WNT signaling during granulosa cell differentiation from squamous to cuboidal and PFA (293, 294). Mouse studies have also suggested that the TNFα signaling through the receptor TNFR2 and downstream nuclear factor κ-light-chain-enhancer of activated B cells (NF-κB) pathway would be key positive regulators of PFA. Loss of Tnfα or Tnfr2 delays PFA (295, 296). This is similar to mutant mice resistant to proinflammatory stress-induced NF-κB activation, which have a larger primordial follicle pool postnatally (297). These data also correlate with a meta-analysis of 22 human genome-wide association studies that identified NF-κB signaling as being strongly associated with the timing of menopause in women (298).

As well as the mechanisms outlined, it should be highlighted that an additional level of regulation exists via noncoding micro-RNAs (miRNA) that likely affect each stage of follicle development, including PFA. These small molecules regulate gene expression at the posttranscriptional level and are conserved across animal species (299). There is an increasing body of work detailing the expression of miRNAs in the ovaries of several species including humans using bioinformatics (300), and their potential use for treating various ovarian conditions particularly POI is being investigated, as reviewed in Ref. 301. There is, however, considerable heterogeneity in the results of studies investigating miRNAs and other noncoding RNAs in human disease, such as in polycystic ovary syndrome (302), and while we acknowledge the importance of these molecules in ovarian/oocyte development, there is still much to clarify. Thus their contributions are not discussed here in detail but have been recently reviewed in the context of the human ovary (301, 303).

PFA is a complex process with more than 1,000 genes being differentially expressed in the oocyte and granulosa cells during the human primordial-to-primary follicle transition (199, 200, 269). Single-cell sequencing techniques are identifying an increasing number of factors involved in regulating the formation and utilization of the primordial reserve in mice, as reviewed in Ref. 304. The mechanisms regulating PFA involve the different signaling pathways interacting with each other (290, 305, 306), forming intricate networks to balance inhibitory and stimulatory signals ensuring the long-term sustainability of the ovarian reserve. Unraveling the role of these factors and the mechanisms that control the selective activation of certain primordial follicles at any given developmental stage remains a major task in the field. While in most cases there is limited evidence as to whether interference with these pathways results in ovarian dysfunction or toxicity in humans (307), their potential importance is of rapidly increasing clinical relevance due to the development and growing use of drugs that target them in the treatment of cancer and other diseases.

## 8. FOLLICLE DEVELOPMENT

A reduction in primordial follicle numbers occurs throughout life with attrition being greatest during prepubertal ages. Activation of follicles begins as soon as the follicle population is formed and follicle growth increases prepubertally (308, 309). AMH, as a marker of the small growing follicle pool, increases through childhood with a plateau observed following the onset of puberty (105, 310). The human ovary shows marked differences in the follicle population over the course of childhood and pubertal development (311). A population of what appear to be abnormal oocytes within primordial follicles is observed prepubertally, and these are eliminated as puberty approaches and progresses, with these abnormal follicles not found in the adult ovary (311). Recent studies have observed differences in histone modifications and chromatin configuration in prepubertal human oocytes compared to adults (312). These differences in follicle populations in the prepubertal human ovary may be analogous to the observations in mice that there are two waves of follicle activation with the prepubertal wave eliminating abnormal follicle structures (313, 314).

**FIGURE 5.**
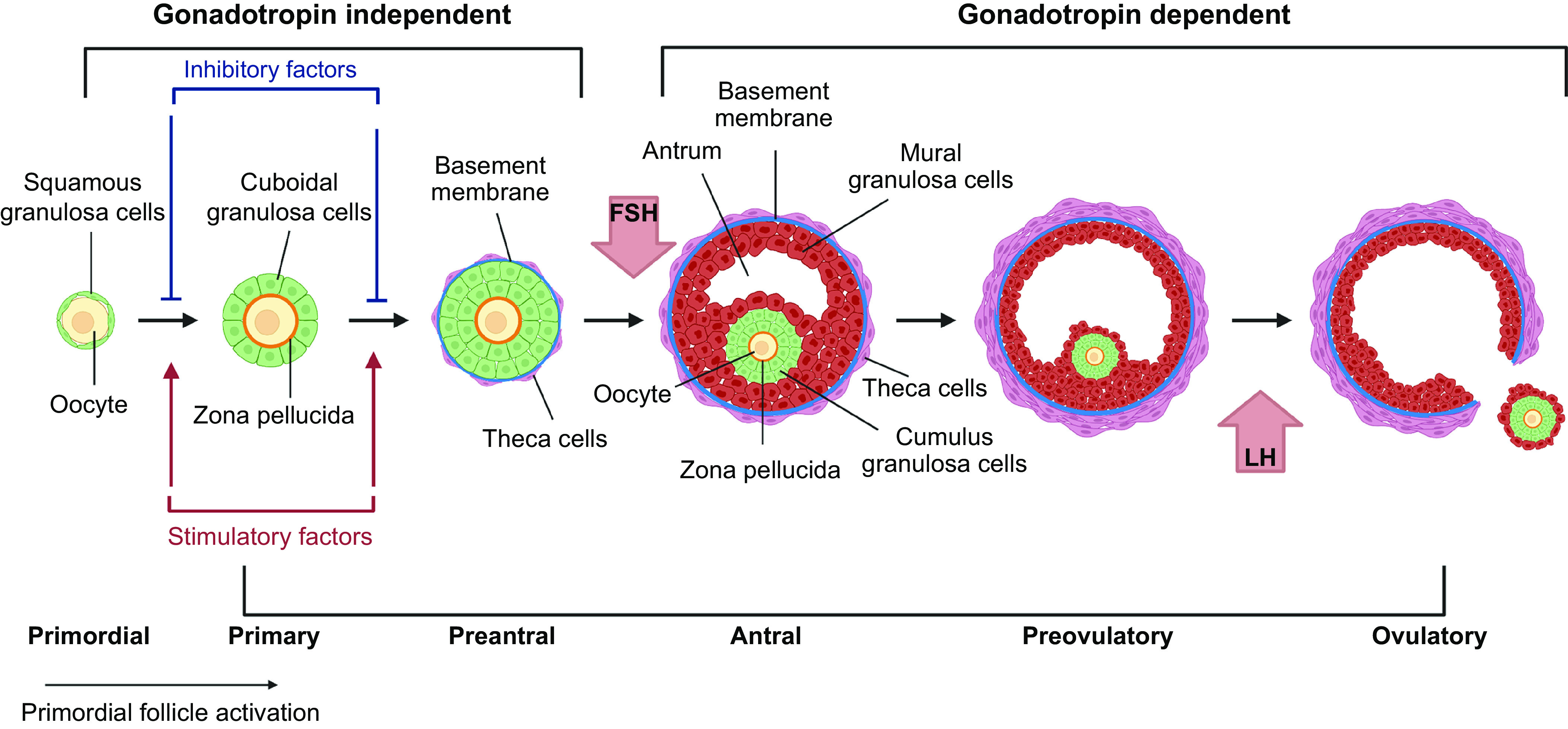
Stages of follicle growth (primordial to preovulatory). Primordial follicles are activated grow to the primary stage which is characterized by the oocyte being surrounded by a complete layer of cuboidal granulosa cells. Under the regulation of paracrine factors, granulosa cells proliferate to form multilaminar structures (preantral), which have differentiated thecal cells organized out with the basement membrane. Follicles then form a fluid filled cavity (antral) with mural granulosa cells lining the wall of the follicle and cumulus granulosa cells surrounding the oocyte. Antral follicles undergo rapid growth to reach preovulatory stages with the oocyte-cumulus complex being released at ovulation in response to luteinizing hormone (LH) signaling. Early stages grow independently of the gonadotropin follicle-stimulating hormone (FSH), but multilaminar stages are acutely dependent on FSH for further growth. Image created with BioRender.com, with permission.

Stages of human follicle development are classified as primordial, secondary, antral, preantral, and preovulatory follicles according to size and number of cell layers (315) (FIGURE 5). The early stages of follicle development are primarily regulated by paracrine factors produced by surrounding somatic cells and nearby growing follicles within the local environment (reviewed in Refs. 316, 317). Later stages, from multilaminar, become increasingly sensitive to and then acutely dependent on the gonadotropins luteinizing hormone (LH) and follicle-stimulating hormone (FSH) (FIGURE 5). The activation of the neuroendocrine axis at puberty provides the support for further growth and hormone production of follicles under stimulatory control from gonadotrophin-releasing hormone from the hypothalamus of the brain through increased secretion of luteinizing hormone and follicle-stimulating hormone from the pituitary (318, 319).

Follicle development has the dual role of *1*) nurturing the oocyte to achieve developmental competence to be fertilized and support embryo development (often termed nuclear and cytoplasmic maturation, respectively), and *2*) producing the sex steroids needed to support the menstrual cycle, endometrial function, and the establishment of pregnancy.

**FIGURE 6.**
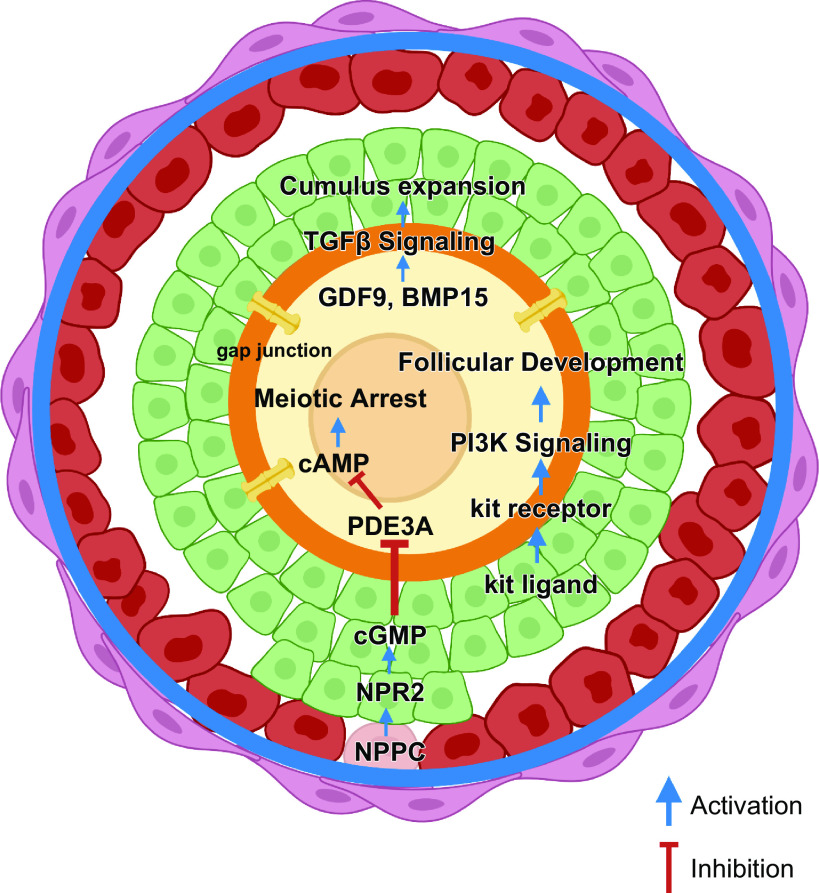
Bidirectional communication within the follicle. Communication between all cell types (oocyte, cumulus and mural granulosa cells and theca) within the growing follicle is facilitated through, gap junctions, transzonal projections (TZPs), and paracrine factors. This communication network is key to maintaining meiotic arrest through maintaining elevated levels of cAMP, facilitating movement of paracrine factors from the granulosa cells to the oocyte (e.g., kit ligand) and oocyte secreted factors (e.g. GDF-9, BMP-15) that affect follicle development (see text). Image created with BioRender.com, with permission.

During follicle development oocytes need to undergo several processes to become developmentally competent: *1*) undergo substantial growth with an increase in diameter from ∼20 µm to 110 µm (human); *2*) acquire competence to resume and complete meiosis; and *3*) acquisition of developmental competence, i.e., capacity for fertilization and formation of embryos. All of these processes are critically dependent on intercellular communication between the growing oocyte and the developing granulosa cells and therefore support and maintenance of these connections are essential as follicles progress through each developmental stage (92) (FIGURE 6). While the follicle creates a microenvironment regulated by paracrine factors that supports oocyte development, external factors such as the ovarian environment and endocrine factors affect these processes as the follicle grows. Mechanical forces imposed by the local ECM and interpreted by ovarian follicles through mechanosensing contribute to regulating the balance between follicular quiescence, activation, and development. It is believed that the dense, collagen-rich cortical region provides a rigid environment that maintains quiescence, while the more pliant medulla layer offers a softer environment that enables follicle expansion and growth (320, 321). Indeed, in vitro studies of isolated murine and primate follicles grown in alginate hydrogels of varying concentrations have confirmed that a stiff environment is necessary to maintain primordial follicle quiescence and survival but negatively affects secondary follicle growth, steroid production, and meiotic potential (322–324). Moreover, follicle spatial distribution within the ovary is uneven and follicular growth follows a geographically determined pattern, shifting from the cortex toward the medulla as folliculogenesis progresses (320, 325).

### 8.1. Preantral and Early Antral Follicle Development

The transition from primordial to primary follicles is characterized by the differentiation and proliferation of granulosa cells to form a single layer of cuboidal-shaped cells that surround the oocyte. Subsequent development through the secondary stage is considered a protracted phase of development to accommodate oocyte growth, and during this proliferative phase, granulosa cells increase to six or seven layers in the preantral follicle (326–328) (FIGURE 5). At this stage, granulosa cells begin to express FSH receptors (183), but they are not dependent on it for growth and differentiation (329), and early stages of follicle development occur independently of gonadotropins (183) (FIGURE 5). During this phase, the oocyte becomes surrounded by the zona pellucida as granulosa cells and the oocyte secrete mucopolysaccharides to form this thick layer of glycoproteins and proteoglycans situated between the oocyte and the granulosa cells (330).

In parallel, the oocyte enlarges and becomes highly transcriptionally active. Some transcripts are immediately translated to support oocyte growth, while others, essential for future maturation and fertilization, are stored for later translation (331). To ensure synchronized development, dynamic bidirectional communications are established between the germinal and somatic cell compartments, manifested either via physical connections or through the secretion of autocrine and paracrine growth factors (332). This dialog allows granulosa cells to support oocyte growth (333) and meiotic progression (334) and to modulate their transcriptional activity (335). In turn, oocytes control granulosa cell proliferation and differentiation into steroid-secreting cells (336) (FIGURE 6).

At the onset of follicular growth, the oocyte and granulosa cells are closely apposed and initiate intimate intercellular connections, developing several cytoplasmic projections and microvilli that interdigitate to generate a large interface for diffusion (337). The zona pellucida is an essential component of the communication network within the developing follicle and once it is formed, narrow cytoplasmic filopodia-like extensions, known as transzonal projections (TZPs), extend from the granulosa cells and traverse the zona pellucida to reach the oocyte plasma membrane (338–340) (FIGURE 6). Located at the tips of the TZPs are gap junctions, intercellular membrane channels composed of connexin proteins that permit the diffusion of mRNAs, ions, metabolites, energy substrates, and signaling molecules up to ∼1 kDa in size between adjacent cells (341). The crucial role of gap junctions during early folliculogenesis has been highlighted by the use of genetically modified mice. Deletion of gap junction protein alpha 4 (Gja4), encoding for the gap junction connexin 37 proteins, completely blocks oocyte-granulosa cell pairing, which halts follicle development at the preantral stage and prevents oocytes from becoming competent (342–344). Ablation of connexin 43 by Gja1 knockout leads to a complementary phenotype: oocyte-granulosa cell coupling is maintained but the communication between granulosa cells is abolished, and folliculogenesis cannot proceed beyond the primary stage (344, 345). These findings reinforce the importance of well-coupled and functional gap junctions between oocyte and granulosa cells and between granulosa cells themselves that maintain the follicle in a functionally integrated state and will ultimately determine oocyte quality. The zona pellucida is a key stabilizing component of this syncytium and its absence leads to infertility (340).

Communication between the oocyte and somatic cells is bidirectional and essential for normal follicle development (346). In parallel to the establishment of physical contacts, once recruited, the oocyte starts to secrete members of the TGF-β family such as GDF9 and BMP15. These oocyte factors directly affect the formation of TZPs as well as granulosa cell proliferation and expansion and trigger the primary/secondary transition (92, 336, 347, 348). GDF9 has been shown to promote the development of human primordial follicles to the secondary stage and to improve follicular survival (349), to control steroidogenesis (350), and to regulate genes involved in cumulus cell expansion (351). A recent report identified a variant of the human GDF9 gene in siblings with POI suggesting an association (352), and low levels of GDF9 and BMP15 in follicular fluid have been associated in young patients with poor in vitro fertilization (IVF) outcomes (353).

BMP15 regulates the early steps of follicular growth closely linked to granulosa cell proliferation (354, 355) and, in later stages, modulates FSH-dependent granulosa cell cytodifferentiation (356), controls cumulus metabolism and expansion (357, 358), and increases oocyte developmental competence (359).

Landmark studies have demonstrated that homozygous loss of function mutations of *GDF9* in mice and sheep (347, 360) and of *BMP15* in sheep (361, 362) results in infertility, with follicles arrested at the primary stage, although mice lacking *BMP15* expression remain fertile (363). Furthermore, aberrant follicular development with impaired fertility has been reported in sheep and cattle immunized against GDF9 and BMP15 (364, 365). In humans, a decrease in *GDF9* mRNA expression has been observed in oocytes from women with PCOS (366), and mutations of *GDF9* and *BMP15* genes contribute to POI (355, 367, 368), reinforcing the key role of these oocyte-secreted factors on ovarian function and fertility. GDF9 and BMP15 also interact with the granulosa-cell secreted factor KL, which is repressed by GDF9 and induced by BMP15 (369).

Another TGF-β superfamily member known to be involved in preantral follicle development is activin (370–372). Activin is produced by both oocyte and granulosa cells and is composed of a dimer of two β-subunits, A or B with activin A being the most prevalent isoform. During folliculogenesis, activin stimulates FSH production from the anterior pituitary (373). Its intraovarian properties comprise increased aromatase activity, antral cavity development, and increased granulosa cell proliferation (370, 374). Activin activity promotes preantral follicle growth in vitro in humans and has been shown to be important in maintaining oocyte-somatic cell communication (375–378) and increasing granulosa cell adhesion to the basement membrane and zona pellucida (378).

Beyond its role as an initiator of PFA, in vitro studies have demonstrated the crucial involvement of the KL/c-KIT system to support a coordinated growth of the follicular complex (203, 379). Moreover, c-Kit- or KL-mutant mice display variable phenotypes ranging from normal fertility to complete sterility associated with arrested follicular development (380, 381), and blocking the c-kit receptor also disturbs the onset of follicular recruitment, primary follicle growth, antrum formation, and granulosa cell proliferation (382). Taken together, these data further demonstrate the importance of coordinated growth between the germinal and somatic compartments.

The regulation of KL, GDF9, and BMP15 is involved in a paracrine negative feedback mechanism. KL activation mediated by BMP15 and GDF9 leads to granulosa cell proliferation. Partly grown oocytes secrete BPM15 leading to KL activation in granulosa cells, while fully grown oocytes mainly produce GDF9 resulting in subsequent inhibition of KL expression in surrounding granulosa cells. In response to the accumulating effects of GDF9, BMP15, and KL secretion, granulosa cells actively proliferate and express FSH, estrogen, and androgen receptors that will be more pronounced as follicles develop (383–385). Under the regulation of this complex of paracrine factors, secondary follicles form multiple layers of granulosa cells and the surrounding theca cells differentiate. This is accompanied by angiogenesis leading to the formation of blood vessels, which are required for further follicle development and ovulation (386). From this stage, endocrine regulation is critical for further follicle development. Under the regulation of GDF9, BMP15 and KL granulosa cells express FSH, estrogen, and androgen receptors. Once the multilaminar follicle reaches a certain size, it forms a fluid-filled space (antral cavity), leading to two functionally distinct populations of granulosa cells: those forming the lining of the cavity (mural) and those surrounding the oocyte (cumulus) (FIGURE 5). These cells, while having a common precursor (387), have been shown to have distinct expression profiles for several paracrine factors and receptors (388) and in humans distinct expression of miRNAs, key regulators of gene expression (389). Oocyte-secreted factors regulate the differentiation of these cells (390). The cumulus cells have a role in regulating oocyte maturation via paracrine regulation while the mural granulosa cells play a role in endocrine regulation and the synthesis of estrogens. Cumulus granulosa cells are in direct contact with the oocyte and form the cumulus oocyte complex (COC) that will be released at ovulation. Communication between the oocyte and cumulus cells regulates the process of cumulus cell expansion and oocyte maturation (336).

Before antral formation, several layers of stromal-like cells appear around the follicular basal lamina and differentiate to form theca layers. At this stage, theca cells express LH receptors and steroidogenic enzymes (391). Theca cells are essential components of the developing follicle, providing structural support and acting in combination with granulosa cells to produce the sex steroids within the ovary (392). Theca cells express the key enzymes to facilitate de novo androgen synthesis from cholesterol (androstenedione) regulated by pituitary LH (393), while the enzymes needed to convert androstenedione to 17β-estradiol (E_2_) [aromatase and 17β-hydroxysteroid dehydrogenase (17β-HSD)] are expressed by the granulosa cells regulated by FSH. The combination of these two cell types and two gonadotropins leads to the production of estradiol (394).

Theca layers are characterized as theca interna (closest to the basal lamina) and the theca externa defining the outer layer of the follicle where blood vessels will form. Formation of new blood vessels (angiogenesis) plays an important role in the ovary, and investment of blood vessels within the theca externa differs in individual follicles making angiogenesis a critical regulator in determining the fate of follicles (395). The vasculature is restricted to the thecal cells, as the basement membrane prevents the invasion of vessels into the granulosa cell layers. As follicles develop through the antral stage, their changing metabolic requirements are met by vasculature remodeling (396). A major regulator of follicular angiogenesis is vascular endothelial growth factor (VEGF), a family of proangiogenesis factors with VEGF-A being the most prominent in the ovary (395, 397). VEGF is expressed in the theca and granulosa cells of secondary follicles and is regulated by gonadotropins at the antral stage. Inhibition of VEGF blocks angiogenesis in thecal cells and ultimately affects follicle development (386).

### 8.2. Follicle Selection to Ovulation

It is clear that the fate of each follicle is influenced by a myriad of factors and that the majority are destined to degenerate. Once follicles reach the antral stage of development, they are acutely dependent on endocrine factors and in particular gonadotropins. The majority of follicles that reach this stage will undergo degeneration and this loss is termed atresia. While primordial follicle death appears to be triggered by the oocyte itself (similar to pathways in fetal life) (143, 398), loss of growing follicles, particularly larger late preantral, antral, and preovulatory stages, is generally triggered by granulosa cell death (141, 398–401). As atresia progresses, the somatic cells detach leading to the antral cavity collapsing and degeneration of the entire follicle.

**FIGURE 7.**
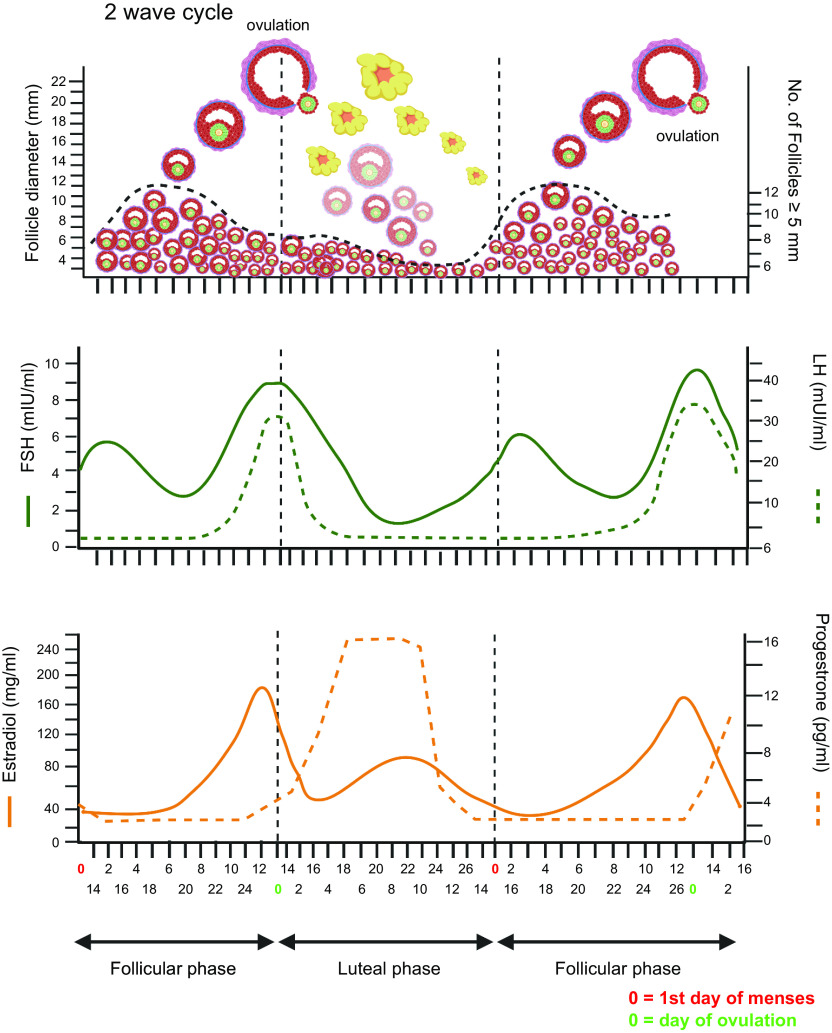
Follicle waves and ovulation. Adapted from Ref. 402, with permission from Oxford University Press. *Top*: illustration of the emergence of a wave of antral follicles and selection of a dominant follicle that survives decreasing levels of follicle-stimulating hormone (FSH) and can respond to the surge of luteinizing hormone (LH) leading to ovulation. Image created with BioRender.com, with permission.

Antral follicles 2–5 mm in diameter are found throughout the ovarian cycle (402, 403). This observation led to the concept that follicles are recruited from the antral population before being selected to progress toward the preovulatory stage. Three models of recruitment from the antral population have been proposed (reviewed in Ref. 404): *1*) continuous recruitment, where antral follicles between 4 and 6 mm in diameter are recruited to grow throughout the menstrual cycle independent of the gonadotropins FSH and LH and the dominant follicle is selected on the basis of it being in the right place at the right time (405); *2*) cyclic recruitment, a cyclical increase in antral follicles between 2 and 5 mm recruited from a continuous supply of growing preantral follicles, which is referred to as secondary recruitment to distinguish it from PFA (406); and *3*) follicular waves, the idea that synchronous growth of a cohort of antral follicles (2–5 mm) occurs at regular intervals has been described as waves. Waves of follicle development have been identified using transvaginal ultrasonography in conjunction with endocrine profiling, and 2–3 waves of 4–14 follicles were detected in healthy women, although not in all (407). This concept of recruitment from waves of antral follicles has been well described in other mono-ovular species such as cow (408) and the mare (409), and modeling/simulation of human parameters support their presence in women (410). A single dominant follicle emerges from the recruited waves to undergo development to the preovulatory stage while the remainder regress (315, 407, 411). Selection takes place during the early to midfollicular phase of the ovarian cycle ultimately leading to ovulation (412) (FIGURE 7).

Whatever the dynamics of selection, the dominant follicle is characterized by its higher production of estradiol than other follicles in the cohort (413, 414). Estradiol produced by the dominant follicle results in negative feedback on pituitary FSH secretion leading to a reduction in FSH and inhibition of the growth of subordinate follicles (408). During the selection process, the dominant follicle becomes more responsive to LH and less dependent on FSH (415). At this stage, the follicle enters an exponential growth phase increasing in size from 5 to 20 mm and the oocyte reaches its mature size of 120 microns and acquires developmental competence.

## 9. MEIOTIC ARREST AND MATURATION

Once oocytes enter prophase I of meiosis and are surrounded by granulosa cells to form primordial follicles, they must be held in meiotic arrest until they receive signals to ovulate (FIGURE 6). While it has been known for some time that this involves the maintenance of elevated cyclic AMP (cAMP) within the oocyte and on somatic cell support, it has taken several decades of research to unravel the regulation of meiotic arrest and maturation in mammalian oocytes. There is now a large body of work, particularly on mouse oocytes, that has demonstrated the regulation of these processes, and it is now known that meiotic arrest involves several cell mechanisms and the interaction of all cell types within the follicle.

Oocytes within the early stages of follicle growth cannot resume meiosis and acquire this ability during follicle growth with meiotic competence achieved before antral formation (416, 417). Following the acquisition of meiotic competence, oocytes need to be held in meiotic arrest until they receive maturation signals at ovulation or they undergo degeneration. The acquisition of meiotic competence is determined by the oocyte acquiring maturation-promoting proteins, cyclin-dependant kinase 1 (CDK1), and cyclin (418–420) to form maturation-promoting factor (MPF; a heterodimer composed of (CDK1) and cyclin B (B1, B2, and B3). Reinitiation of meiosis requires the activation of MPF (421), and during oocyte growth, these proteins (cyclin B1 and CDK1) accumulate and acquire an increased ability to combine (419).

As the oocyte’s capacity to produce MPF increases, its activation needs to be inhibited to prevent the premature resumption of meiosis, which has been known for some time to be dependent on cAMP (422). When intracellular cAMP levels are elevated in the oocyte, activation of MPF is inhibited through the action of cAMP-dependent protein kinase A (PKA) (423, 424). Activation of PKA results in the phosphorylation and activation of nuclear kinase Weel/MytI, which inactivates the activator of cyclin-dependent kinase, cell division cycle 25B (CDC25B), leading to the phosphorylation of CDK1 being inhibited, thus making MPF inactive and so holding the oocyte in meiotic arrest.

Given that removal of the oocyte from the follicle results in the spontaneous resumption of meiosis (425, 426), it was assumed that the source of cAMP was produced in the somatic cells and transported to the oocyte via gap junctions (427). However, experiments in mice where gap junctions were experimentally closed to prevent any transfer of factors showed that cAMP levels did not fall dramatically, indicating that the oocyte was capable of producing cAMP independently of somatic cells (428).

It has now been established that cAMP can be produced by the oocyte and high levels are maintained via the stimulating G protein (G_s_) signal transduction pathway. Synthesis of cAMP is from ATP via adenylyl cyclase (AC) and is degraded by phosphodiesterases (PDEs). In rodent oocytes, a constitutively active G-protein coupled receptor 3 (GPR3) has been located on the oocyte membrane (oolema) and has also been identified in human oocytes (429). GPR3 leads to the activation of AC by G_s_ and cAMP production (416, 430–433). Two distinct PDE isoenzymes (PDE4 and PDE3A) are expressed within the ovarian follicle (for review, see Ref. 434). PDE4 is expressed in granulosa and theca cells while PDE3A is expressed exclusively in the oocyte (435).

While it is now accepted that oocyte production of cAMP is the major pathway in regulating meiotic arrest, somatic cells also have an indirect role in maintaining elevated cAMP levels within the oocyte via cGMP from the granulosa cells inhibiting PDE3 activity within the oocyte. This is mediated via the natriuretic peptide C/natriuretic peptide receptor 2 (NPPC/NPR2) system. C-type natriuretic peptide (CNP) is produced in mural granulosa cells, and its receptor NPR2 (a member of the guanyl cyclase receptor family) is expressed within cumulus granulosa cells. The production of cGMP in the granulosa cells inhibits the degradation of cAMP by inhibiting PDE3 activity in the oocyte (436). The regulation of this pathway involves communication between mural and cumulus granulosa cells and bidirectional communication with the oocyte (FIGURE 6).

### 9.1. Ovulation and Oocyte Maturation

The LH surge triggers ovulation and stimulates a sequence of events in the ovulatory follicle leading to the resumption of meiosis in the oocyte, mucification, and expansion of the cumulus, and rupture of the follicle resulting in the release of the cumulus-oocyte complex (COC) into the fallopian tube. Once the COC is released, the granulosa and thecal cells become luteinized and form the corpus luteum.

The resumption of meiosis to metaphase II is initiated by the surge of pituitary LH that occurs midcycle and triggers ovulation. LH stimulates oocyte maturation via its action on the theca and granulosa cells (437) and indirectly on the oocyte via the expression of genes regulating EGF, which influences nuclear maturation and oocyte competence (438). The EGF network is not discussed in detail here but has been reviewed by Richani and Gilchrist (439).

LH binds to the LH receptor (LHR) a G protein-coupled receptor (GPCR) expressed on the membrane of mural granulosa cells and theca cells (440, 441). However, the oocyte does not express LH receptors and so the action of LH is mediated via members of the epidermal growth factor (EGF)-like family, in particular amphiregulin (AREG) and epiregulin (EREG) (438) whose production is upregulated. AREG and EREG are synthesized as precursors within the oolemma their extracellular domains are cleaved by the transmembrane metalloprotease ADAM17/TACE (442) to release the active forms. These peptides induce changes in mural and cumulus granulosa cells (438, 443) that influence nuclear maturation and oocyte competence (438).

**FIGURE 8.**
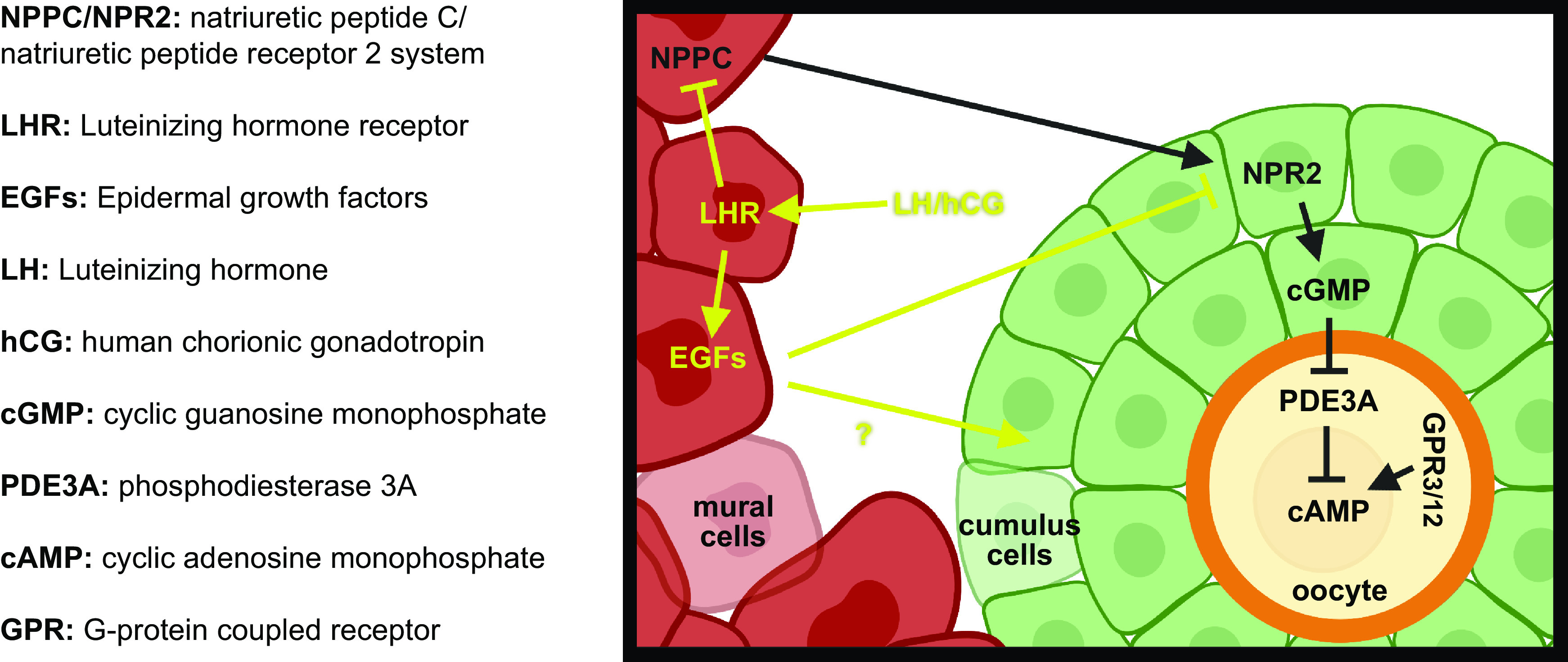
Meiotic activation. Resumption of meiosis and ovulation is triggered by luteinizing hormone (LH). LH signaling downregulates the NPPC/NPR2 system causing reduction in cAMP and cGMP levels within the oocyte leading to the phosphorylation of PDE3A and the degradation of cAMP triggering the resumption of meiosis and driving the formation of the first meiotic spindle. Image created with BioRender.com, with permission.

LH signaling reduces the cAMP level within the oocyte through downregulating the NPPC/NPR2 system (444) and closing gap junctions between the oocyte and cumulus cells (445, 446). Intercellular communication within the follicle is disrupted (447) via its effect on the translation of Cx43 (connexion-43) protein (448) and activation of a mitogen-activated protein kinase (MAPK)-dependent pathway resulting in phosphorylation of connexin-43 (449). Cx43 is the dominant connexin connecting gap junctions between granulosa cells and granulosa cells whereas CX37 is the main connexion connecting cumulus granulosa and the oocyte. As discussed above, meiotic arrest is maintained via elevated cAMP and cGMP levels. By affecting the NPPC/NPR2 system and disrupting cell-cell communication, cAMP and cGMP levels are reduced within the oocyte leading to the phosphorylation of PDE3A and the degradation of cAMP (FIGURE 8). This sequence of events results in the synthesis of MPF and phosphorylation of proteins such as APC leading to the resumption of meiosis and driving the formation of the first meiotic spindle (450). The activated CDK1-cyclin B complex phosphorylates downstream substrates including spindle assembly checkpoint (SAC) proteins (451). These SAC proteins ensure the normal progression of the cell cycle and segregation of chromosomes (452).

**FIGURE 9.**
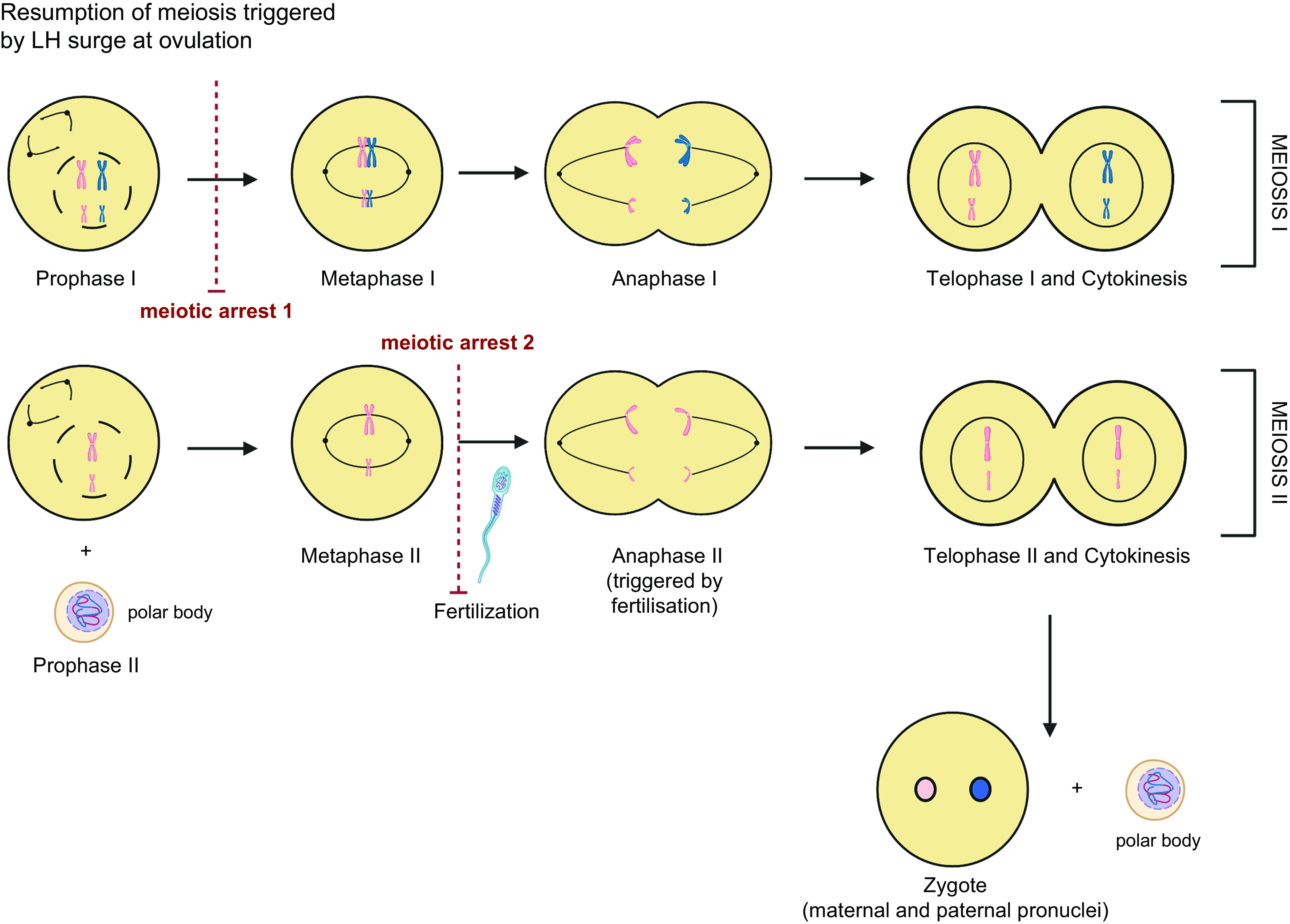
Stages of meiosis. As oocytes are formed during fetal life they enter meiosis, reach the dictyate stage of prophase 1, and are then held in meiotic arrest. Meiosis resumes in response to the luteinizing hormone (LH) surge at the time of ovulation and progresses through the first meiotic division with emission of the first polar body and the formation of the metaphase II spindle and arrested for a second time. Final resumption and completion of meiosis is triggered by fertilization. Image created with BioRender.com, with permission.

The first morphological sign that meiosis has resumed is that the nuclear envelope [germinal vesicle (GV)] breaks down (GVBD). This marks the start of metaphase I with compaction of chromosomes and the homologs orient on the metaphase I plate and segregating at anaphase I leading to the extrusion of one set of chromosomes in a small bleb of cytoplasm, i.e., the first polar body (PB1) (FIGURE 9). This first division is an even division of chromosomes but uneven of cytoplasm. Following this division, a second spindle forms, and the chromosomes align on the spindle. Meiosis is arrested for a second time at metaphase II and will remain arrested until fertilization or degeneration. Fertilization initiates the resumption of meiosis and the separation of sister chromatids (FIGURE 9).

### 9.2. Cytoplasmic Maturation

Cytoplasmic maturation indicates the molecular and cytoskeletal changes as well as organelle reorganization and other processes that prepare the oocyte for fertilization and preimplantation development. Just as competence to undergo nuclear maturation is acquired in a stepwise manner during follicle development so is cytoplasmic maturation. Defining the process of cytoplasmic maturation remains vague despite the search for markers of cytoplasmic and developmental competence (453).

As oocytes grow through the stages of follicle development, they are transcriptionally active, producing the mRNAs required to support oocyte maturation and early embryonic development. Before ovulation oocytes are transcriptionally repressed. The coordinated changes that take place in organelle and cytoskeletal structure necessary for maturation are regulated through the translation of prestored RNA in the oocyte and RNA binding proteins. Key to driving these processes is the maternally derived mitochondria that provide energy to support nuclear and cytoplasmic maturation (454).

With each ovarian and menstrual cycle, normally one oocyte will be ovulated and either fertilized or degenerated. With time, there is a progressive diminishing of the pool of follicles/oocytes and the aging process impacts on oocyte quality. As well as changes in oocyte number and quality, several other changes occur in the aging ovary.

## 10. OVARIAN AGING

The changes in the ovary that accompany aging are of increasing importance both in society and clinically. There has been a consistent rise in age at motherhood across many developed societies resulting in many women not starting their families until they are in their thirties, which translates clinically into age having an ever-growing importance in the prognosis for many patients attending for medical assistance in conceiving. Women tend to have slightly older male partners, and while the impact of age on male fertility is much less than on women, it can also have an impact (455). While female fertility declines markedly across a woman’s thirties, clearly reflected in the declining success of assisted reproduction treatments, ovarian function continues for approximately a decade after the loss of fertility until menopause. The amenorrhea of menopause reflects a lack of sufficient estrogen production by growing follicles to cause endometrial stimulation and is often preceded by menstrual irregularity as the previously consistent steady stream of growing follicles becomes erratic.

**FIGURE 10.**
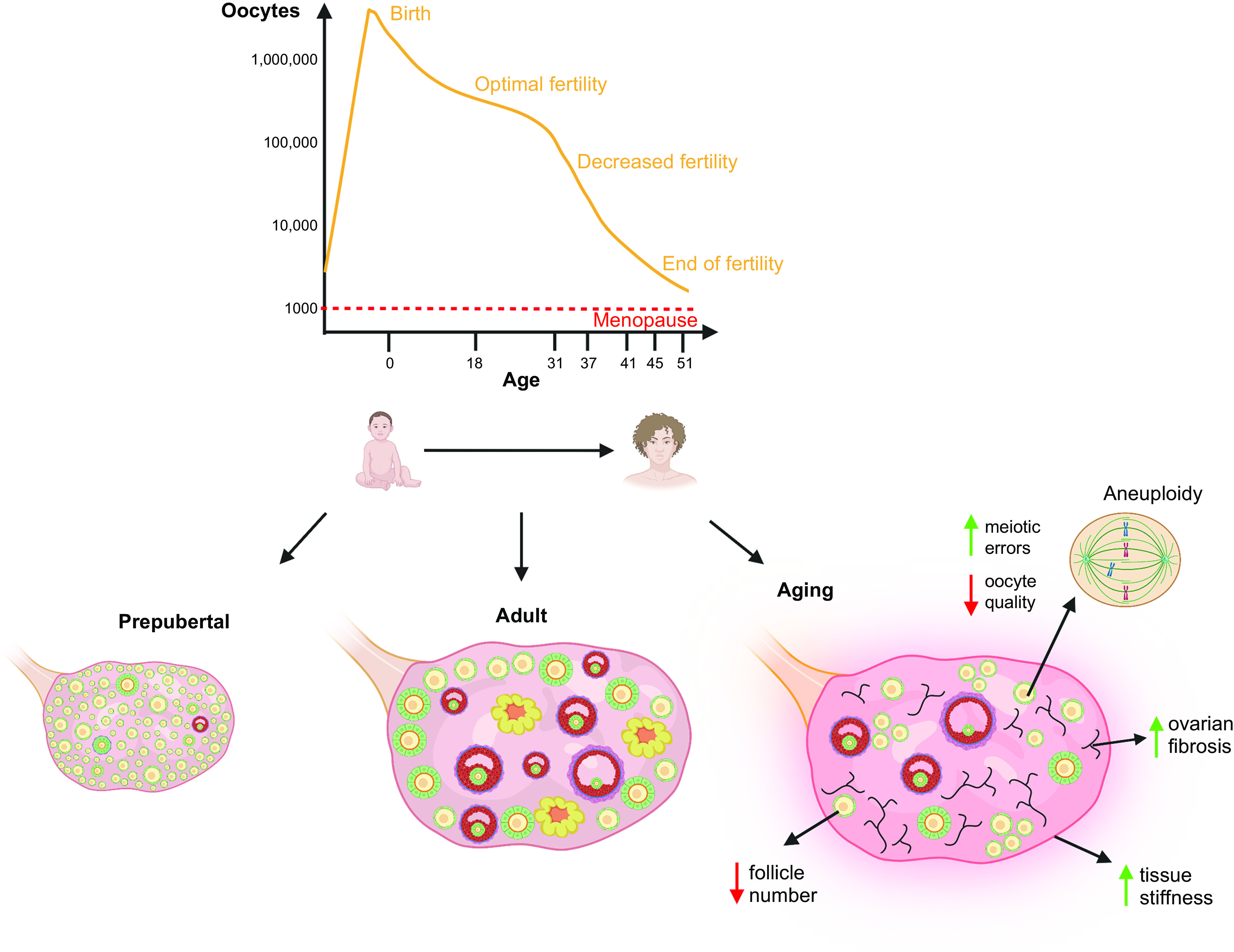
Representation of the key changes in the ovary with age. Prepubertally the ovary has a maximum endowment of follicles, with follicle growth present to early antral stages. After puberty, all stages of follicle development, ovulation, and corpora lutea are present. In later reproductive life, there is depletion of the primordial and growing follicle pool but ovulation continues until the menopausal transition. There is also an increasingly uneven distribution of primordial follicles with some clustering, and increasing fibrosis of the stroma affecting its mechanical properties. Image created with BioRender.com, with permission.

This in turn reflects one of the two key aspects of ovarian aging, which is the incessant decline in the number of primordial follicles, the ovarian reserve, across life. Despite the clinical difficulties in counting nongrowing follicles in a large structure such as a whole human ovary and the inevitable scarcity of such samples, a number of models have been developed over the years to examine this decline. Initial analyses by Faddy and Gosden (456, 457) and Gougeon (458) identified an accelerated rate of loss in later reproductive years, i.e., the later thirties, consistent with a broken stick model (FIGURE 10). Subsequent analyses have suggested a more biologically plausible continuously increasing rate of loss (459, 460) (albeit based on small datasets), which was also identified in the most recent version of this modeling, based on a combination of databases (106). These approaches are essentially mathematical, based on deriving a best fit to the experimental data. An alternate analysis has recently been performed based on modeling fluctuation in the activity of the ISR (a “random walk”) with a nonrandom drift toward lower activity supporting follicle activation over time (461). Using biological data only of the number of primordial follicles at birth, this mechanistic approach generates follicle number decay curves in close agreement with the above-mentioned mathematical models using biological data at all ages and also provides a distribution of age at natural menopause that approximates clinically derived data.

While recognizing the substantial variation between individuals, analysis identifies that the number of nongrowing follicles in the ovary halves approximately every 5 years from the midtwenties (106). Variation between women on the fifth and ninety-fifth percentile for follicle number means that the loss is ∼20 a month at age 35 in those with fewer follicles, versus 200 a month at the same age in those on the ninety-fifth percentile. As this is the number of follicles leaving the nongrowing pool, either to initiate growth or to become immediately atretic, this provides a clear exposition of why some women will approach menopause at young ages as a consequence of being at the extreme of the range of follicle endowment, with significant consequences for their fertility. This late acceleration of follicle depletion has been recognized for at least 35 years (462), but as described above it is of growing clinical importance. There are also changes in the distribution of the remaining primordial follicles within the cortex of the ovary, with an increasingly uneven distribution. Thus nongrowing follicles are more clustered together (463), presumably reflecting (and perhaps generating) a more inhibitory environment for the initiation of follicle growth. This is consistent with spatial analysis of nongrowing and growing follicles in the mouse ovary (283), which also indicated that nongrowing follicles tended to be nearer each other. This was interpreted as consistent with the local production of growth-inhibitory factors but may also involve local variation in the mechanical characteristics of the stroma (320) (FIGURE 10).

These changes in ovarian function with age are reflected in endocrine changes. A monotropic rise in FSH concentrations has long been recognized as a hallmark of ovarian aging before menopause and has been shown to be due to reduced feedback at the pituitary from lower inhibin B concentration from the reduced number of antral follicles (464, 465). These late stages of follicle development are distant from the primordial pool; thus a biomarker that more accurately reflects that pool would be of great value. Recently most interest has focused on the measurement of AMH as the circulating marker currently available that most closely reflects the nongrowing pool. Although classically a “male” hormone produced by the immature Sertoli cell and its absence critical for prenatal development of the reproductive tract in females, it was subsequently shown to be produced by the granulosa cells of growing follicles (466) and in mice at least to be an important regulator of PFA (272). AMH production only starts in growing follicles; thus it is not a direct marker of the ovarian reserve, although that term is widely used in reproductive medicine to mean the number of follicles that can be stimulated to grow by exogenous FSH administration (also termed the “functional ovarian reserve”). Indeed the most established clinical value of AMH is as a predictor of the ovarian response to FSH in the context of superovulation for assisted reproduction (467). However, initial clinical studies identified a decline in AMH with age (468), and in the context of aging, AMH measurement can identify women with a very low ovarian reserve and thus be used to predict or diagnose menopause (reviewed in Ref. 469), although it is clearly more accurate at excluding imminent menopause than predicting it (470).

While the original endowment of the primordial follicle pool formed in utero (40) is likely to be a key variable in the determination of the established adult ovarian reserve, there has also been much interest in lifestyle and environmental factors that might influence the rate of decline (471). In addition to overtly toxic chemicals such as those used in the treatment of cancer, there is growing literature on the potential adverse effects of a large number of environmental chemicals and lifestyle factors. While cigarette smoking has been postulated to be a factor (472), alcohol consumption also has an appreciable effect, although in that analysis tobacco smoking, oral contraceptive use, and patient’s body mass index (BMI) were not found to have an identifiable effect (473). This remains an important area of ongoing research, however, as there is a clearly validated relationship between the age at natural menopause predicted from the number of nongrowing follicles in the ovary and actual age at menopause (474). The size of the primordial follicle pool is indeed an important determinant of ovarian lifespan and a direct biomarker would be of substantial benefit in research and in clinical practice.

In addition to research on extrinsic influences on remaining follicle number and by implication the function and quality of the growing follicles and oocytes, large-scale genetic studies have also sought to identify key genetic associations with reproductive lifespan. Given the relationship between reproductive lifespan and fertility, such studies can also be seen as pointers toward the genetic regulation of oocyte quality. In addition to important though rare single gene defects (475), recent studies have focused on DNA damage and its repair with a number of genes directly involved in these pathways being implicated at the age of natural menopause (476). This work builds on a previous similar analysis from the same group linking reproductive aging to breast cancer susceptibility through DNA repair, with hypothalamic signaling also linked to these genetic pathways (477). In this recent analysis of 200,000 women, a genetic score for age at natural menopause was generated, with 290 genes associated with that outcome. Strikingly, there was a continuous variation in this genetic score across the range of age at menopause, including in women with early menopause (i.e., under the age of 45) and even in women with overt POI although the numbers of such women included were small. Thus the odds ratio for menopause age under 45 and for POI varied with the percentile of genetic risk. This work brings to the fore the importance of DNA damage and its repair in the oocyte in particular and gives a broader context to previous studies that have investigated ovarian function in women with mutations in a DNA repair gene of particular importance, the BRCA1 gene. These studies have shown that BRCA1 mutation carriers have AMH levels ∼25% lower than age-matched noncarriers and were more likely to have AMH concentrations in the lowest quartile for their age, with an odds ratio of 1.8 (478). This has been confirmed in a recent meta-analysis, also showing that this reduction does not seem to be the case for women who have a mutation of BRCA2 (479). Women with BRCA1 mutations also show a smaller number of oocytes obtained after ovarian stimulation with a markedly increased risk of a poor response, with an odds ratio for that of 38.4 (confidence intervals 4.1 to 353) (480). While the implications of variations in DNA damage and repair function in granulosa cells are less clear, there is evidence from nonhuman primates that there is also a loss of DNA damage repair capacity in granulosa cells with increasing age (481). This also has clinical implications during medically assisted reproduction, as the reduced DNA capacity of oocytes from older women is considered less able to contribute to DNA repair in sperm.

## 11. OOCYTE QUALITY AND AGE

In addition to the major changes in follicle/oocyte number with age, there are also changes in oocyte “quality” that have a major impact on female fertility and the success of assisted reproduction. The importance of oocyte quality, i.e., the potential to develop into a normal pregnancy and ultimately a heathy baby, has long been recognized. Classic studies have documented the falling success rate of IVF with age and how that did not occur when the oocytes used were donated from young women (482), and the rising probability of a pregnancy ending in miscarriage (483). In keeping with this, screening programs for detecting pregnancies affected with Down syndrome (due to trisomy 21) and other chromosomal abnormalities were initially based solely on maternal age, although this is now supplemented with measurement of biomarkers in the blood and by ultrasound.

The key basis for this decline in oocyte quality with age is the increased risk of chromosomal aneuploidy. Meiosis is initiated in fetal life, with oocytes progressing through the early stages of meiosis I before arresting in the diplotene stage of prophase. This includes the process of chromosomal crossover (recombination) after pairing of homologous chromosomes, through which genetic material is exchanged between the parental chromosomes. This is then maintained until meiosis resumes at the time of ovulation, triggered by a cascade of biochemical changes initiated by the LH surge (484). In women, this means that meiotic arrest is maintained for an extraordinarily long period of time, over several decades. Completion of meiosis I involves the separation of homologous chromosomes to opposite poles of the meiotic spindle (and thus one set into the first polar body), with sister chromatids being retained together at the same pole. Meiosis II involves the orientation of sister chromatids on the meiotic spindle and their subsequent separation, thus also involving cleavage of the centromere, with the passage of one set into the second polar body thus generating a haploid gamete. The sperm chromosomes have already entered the oocyte; two pronuclei form, fusing at the first mitotic division of the zygote (485).

While several mechanisms for oocyte aneuploidy have been identified, including recombination/crossover formation defects, cohesin loss, spindle deformation, spindle assembly checkpoint malfunction, microtubule-kinetochore attachment failure, kinetochore misorientation, and mitochondria dysfunction with increases in reactive oxygen species [detailed in recent reviews (486–488)] with in some cases the specific genetic or cell cycle-related mechanisms identified (489, 490) it is now recognized that it is the cohesion between sister chromatids which is central to the subsequent fidelity of chromosomal allocation to the oocyte versus the polar bodies that underlie the major age-related decline (491). Cohesion, mediated by the number and site of cohesin rings, is established during the S phase of cell division and cannot be repaired or replaced. Over time, cohesion is lost resulting in premature sister chromatid separation and thus an increased risk of the univalent chromatids mis-segregating, resulting in oocyte aneuploidy. Alternative mechanisms of abnormal chromosome segregation also occur, namely nondisjunction whereby homologous chromosomes or sister chromatids do not segregate at either meiosis I or meiosis II, and reverse segregation, whereby one sister chromatid from each of a pair of chromosomes separates at meiosis I: while this results in a euploid oocyte at that stage, correct subsequent alignment is unlikely to occur and meiosis II can result in aneuploidy (492). However, it is premature sister chromatid separation that predominantly increases with age and is the most common mechanism resulting in aneuploidy related to maternal age. Reverse segregation also becomes more common with age, whereas nondisjunction in fact decreases with age (493). This may reflect the high level of cohesion at a young age and underpin the slightly increased risk of aneuploidy in the oocytes of very young women, before it reaches the “sweet spot” that minimizes aneuploidy risk until later maternal age when its progressive loss results in the later rise in risk, though premature sister chromatid separation.

Other aspects of oocyte function that decline with age and impact on spindle arrangement are also being identified, particularly in rodent models but with potential therapeutic importance. The intraovarian levels of several members of the sirtuin family of regulators of mitochondrial function and oxidative stress decline with age (494), and older mice with oocyte-specific knockout of *sirt1* show reduced fertility and oocyte quality without an effect on follicle number (495). This was not seen in younger animals, consistent with redundancy with other sirtuins, but the phenotype was apparent when additional stress, i.e., reduced NAD^+^ concentrations, was added. Conversely, administration of the NAD^+^ precursor nicotinamide mononucleotide (NMN) to older mice improved meiotic spindle assembly and increased both oocyte yield and the number of pups born, with improved mitochondrial function and reductions in oocyte reactive oxidative species (496, 497). Given the degree of interest in NMN as a treatment in many aspects of aging (498), this has immediate clinical applicability if safety issues can be addressed.

Clinically, this increased prevalence of aneuploid oocytes results in an increased risk of miscarriage and pregnancies complicated by chromosomal abnormalities, which depending on the chromosome involved, may or may not result in a live birth. The prevalence of trisomies rises from 2% of pregnancies in young women to over 40% at age 40, and the proportion of aneuploid embryos rises from ∼5% between the ages of 26 and 37 to 33% at age 42 and 53% at age 44 (499). This huge attrition rate cannot be prevented, but its early detection has been an area of great scientific and clinical activity in assisted reproduction, and indeed of controversy (500–502), based on the premise that identification of aneuploid embryos allows for their deselection for transfer to the patient. While this cannot increase the live birth rate from the time of initiation of treatment, by removing abnormal embryos from the pool of those that could be selected for transfer, the chances of selecting one that will result in live birth would be expected to increase. Thus a higher live birth rate per embryo transfer should be achieved and possibly also a reduction in the time interval between starting treatment and achieving a viable pregnancy. These different denominators have contributed to the controversy in the field, with additional arguments put that, for example, for an older woman, knowing that she has no euploid embryos before the transfer can be helpful, as well as avoiding the time and expense of replacing embryos that either fail to achieve a pregnancy or worse, result in miscarriage or other adverse pregnancy outcomes.

The laboratory techniques used to assess embryo euploidy have changed from fluorescent in situ hybridization (FISH) on a blastomere biopsied from a cleavage stage embryo to next-generation DNA sequencing of several cells from a trophectoderm biopsy, generally 5 days after fertilization (503). Preimplantation genetic testing for aneuploidy (PGT-A) as it is now known, previously termed preimplantation genetic screening (PGS), has become very widespread, such that is now the norm in many U.S. IVF clinics, though much less widely used in Europe (504). With the new knowledge derived from such advanced techniques has also come difficulties in interpretation. The most important of these has been the issue of mosaicism (505–507), wherein some of the cells in the trophoblast biopsy appear euploid and others aneuploid, with the unknown aspect being how these reflect the cells of the inner cell mass, which will become the future baby, or how this might affect the pregnancy. Deselecting such embryos for transfer risks denying the possibility of a live birth, and of course undiagnosed mosaic embryos have been and continue to be replaced throughout the history of IVF, without apparent adverse neonatal outcomes. Initial clinical reports on the deliberate replacement of mosaic embryos reported successful normal births, and it is now clear that while mosaic embryos overall have lower developmental potential, this is related to the degree of mosaicism (508, 509), such that when <50% of cells in the biopsy are abnormal, pregnancy outcomes are good.

An additional question of clinical importance is whether the oocytes of women with a reduced ovarian reserve for their age are also of poorer quality, or whether in that situation, quality and quantity are not related. An analysis of 9,489 cycles among 8,214 patients found no association between live birth and ovarian reserve among pregnant IVF patients under the age of 35 years (510), and in a study of oocyte donors, ovarian reserve was not associated with pregnancy rates if at least four oocytes were obtained (511) This was more directly supported in recent studies that also involved PGT-A: analysis of 1,718 women found no differences in the proportion of aneuploid embryos by AMH level and no differences in live birth rate (512). A recent study also found that the proportion of aneuploid embryos was similar in 383 women diagnosed with diminished ovarian reserve compared with matched controls (matched for age, BMI, and cycle number), at 42.2% versus 41.7% (513). Live birth rates per euploid embryo transfer were also similar. Thus oocyte quality and quantity do not seem to be directly linked in younger women with reduced ovarian reserve.

## 12. AGE AND THE OVARIAN STROMA

There is a growing interest in the contribution of the ovarian stroma to ovarian aging and thus to its important role in supporting normal follicle development throughout life. In the human ovary, the stroma is densely packed with spindle-shaped fibroblast-like cells, with a key change during aging being increased fibrosis. The increased collagen content changes the physical properties of the ovary, with increasing stiffness (514). A detailed analysis of what has been termed the mechanical matrisome has recently been published highlighting increased collagen content and reduced elastin in the human ovarian cortex, indicating a stiffer extracellular matrix impacting on the initiation of and subsequent follicle growth (515). Intriguingly, higher collagen and lower elastin content were also identified around primordial and small growing follicles in the prepubertal human ovary, changes that may also have implications for limiting the initiation of follicle growth at that time in life.

In rodent models, increasing fibrosis has also been described in distinct foci initially, accompanied by a population of multinucleated macrophage giant cells, associated with chronic inflammation that is also profibrotic (516). Others have found no increase in fibrin content in the mouse ovary at up to 18 months of age (517): it is unclear whether this is a strain difference (increased fibrosis in CD1 and CB6F1 but not C57BL/6) or another explanation. However, that study also highlighted the increased inflammatory cell population of the mouse ovary with age, with increased numbers of CD4^+^ T cells, B cells, and macrophages as well as increased expression of proinflammatory cytokines and the inflammasome genes ASC and NLRP3 (517). Knockout mouse models of these two inflammasome genes also show some evidence of reduced loss of primordial follicles with age, associated with reduced macrophage numbers within the ovary and reduced expression of inflammatory cytokines (518). The potential importance of immune cell involvement in the ovary has recently been highlighted by the demonstration that immune checkpoint inhibitors increased immune cell infiltration into the mouse ovary and resulted in reduced numbers of both primordial follicles and growing follicle stages, with estrous cycle irregularity and reduced numbers of corpora lutea (519). These drugs are increasingly being used clinically to treat a range of cancers, but no ovarian toxicity studies were performed before licensing approval and only very limited clinical data are currently available (520).

Treatment with the drug metformin is associated with reduced fibrosis in a number of organs, linked to AMPK-mediated stimulation of TGF-β production. This has also recently been described in the ovaries of diabetic women taking metformin (521). In addition to an overall increase in collagen content, collagen fibers become increasingly anisotropic (linearized) with age, whereas they were found to be more isotropic in the ovaries of women taking metformin. In addition to potential consequences for follicle quiescence and growth, this was also interpreted in relation to reduced ovarian cancer incidence, which has been identified in women with diabetes who take metformin, through postulated changes in the protumor niche.

Animal model data support the therapeutic potential of reducing ovarian fibrosis associated with age. Treatment of aged mice (15 months of age) with clinically available drugs used to treat pulmonary fibrosis resulted in a reduction in ovarian cortical collagen content and, in most but not all animals, restoration of the ovarian response to superovulation (522). Remarkably, in the case of one of the drugs, this effect occurred after only 4 days of treatment. Natural ovulation and conception were not assessed. Reduced mitochondrial function was also identified in ovarian stromal cells from aged mice and prevented by antifibrosis drug treatment and the mitochondrial function enhancer BGP-15 (522). Comparable findings were also identified in a mouse model of overeating and obesity, with accelerated fibrosis and ovulatory dysfunction, which was reduced by only 4 days of antifibrosis drug treatment, and, in keeping with the data described above from diabetic women (521), both obese and aged mice exposed to metformin treatment for 2 weeks showed reduced fibrosis, but an increased follicular response to gonadotrophin administration was only seen in the metformin-treated obese mice.

The consequences of age-related increased fibrosis have not been greatly explored, but it is possible that it also plays a role following the recovery of ovarian function following chemotherapy treatment. Such treatment has been demonstrated to increase collagen deposition in the human ovary (523) and thus may be comparable to an aging effect. In an analysis of AMH concentration in women during and following treatment for Hodgkin lymphoma, ovarian function recovery was less complete in older women independent of pretreatment AMH levels. In contrast, younger women showed full recovery of AMH levels, again independent of whether their AMH level pretreatment was low or high (524).

The poor vascularization of the superficial ovarian cortex has related to ongoing quiescence of the primordial follicle pool (525) and the slow growth rate of primary and early secondary follicles. Early vascular alterations with age consist of thickening of the vessel wall in both the ovarian medulla and deeper cortex, which had been identified in relatively young women of thirty years of age and onwards (526, 527). It has thus been proposed that these age-related changes in the ovarian blood vessels occur earlier than in other organs.

It is possible that these changes in the extracellular matrix are in part endocrine related. In mice, LH has been associated with ovarian fibrosis and this was reduced by treatment with a GnRH antagonist (528). These studies were performed using a granulosa cell-specific NRG1 knockout mouse, which is a model of accelerated aging, exhibiting longer estrus cycles and reduced fertility. Increased collagen expression was seen in younger GC-NRG1 knockout mice compared to older wild-type mice and was associated with an increased number of LH receptor-positive endocrine cells in the stroma. Treatment for only 8 days with a GnRH antagonist caused some reversal of this ovarian histology and subsequent improvements in follicular development and indeed in fertility (528). This therefore not only supports the impact of stromal fibrosis on follicular growth but also suggests an endocrine-mediated pathway and a potential for therapeutic intervention. These studies have striking parallels with the typical situation where the use of GnRH analogs to protect the ovaries of women particularly with breast cancer against chemotherapy has been widely investigated. Thus, large RCTs of this approach have indeed confirmed a reduced risk of POI in women cotreated with GnRH agonists during chemotherapy (529), although effects on preservation of later fertility are less clear. The mechanisms of this are unknown, but it is intriguing to speculate that protection against stromal fibrosis may be a component of this effect.

## 13. IN VITRO DEVELOPMENT OF OOCYTES

As detailed in earlier sections, our understanding of the processes and regulation of oocyte/follicle development has been founded on a large body of experimental studies utilizing a range of models that have relied heavily on rodents with extrapolation to humans. While rodent transgenic and knockout models have been essential in elucidating regulatory pathways and mechanisms that may apply across species, there is a need for tractable models to study human-specific processes relating to germline development (7, 530). Basic research on developing in vitro model systems to study oocyte development as well as attempting to create oocytes from pluripotent stem cells has been ongoing in rodents for decades and has contributed to our increased understanding of human oocyte biology. Advances are now being made in developing and utilizing human-specific models, and these will expedite progress in translating basic research to clinical application. Deciphering how human oocyte growth and development are regulated will lead to the emergence of novel techniques for preserving fertility and treating infertility. This section deals with the progress that has been made in developing in vitro systems to support the growth of human oocytes and the formation of new oocytes from pluripotent (both embryonic and induced) stem cells. This is distinct from what is generally termed “in vitro maturation” (IVM) in a clinical context, which solely refers to the final stages of maturation, following the aspiration of essentially fully grown oocytes that have yet to resume meiosis.

### 13.1. Developing Oocytes In Vitro

**FIGURE 11.**
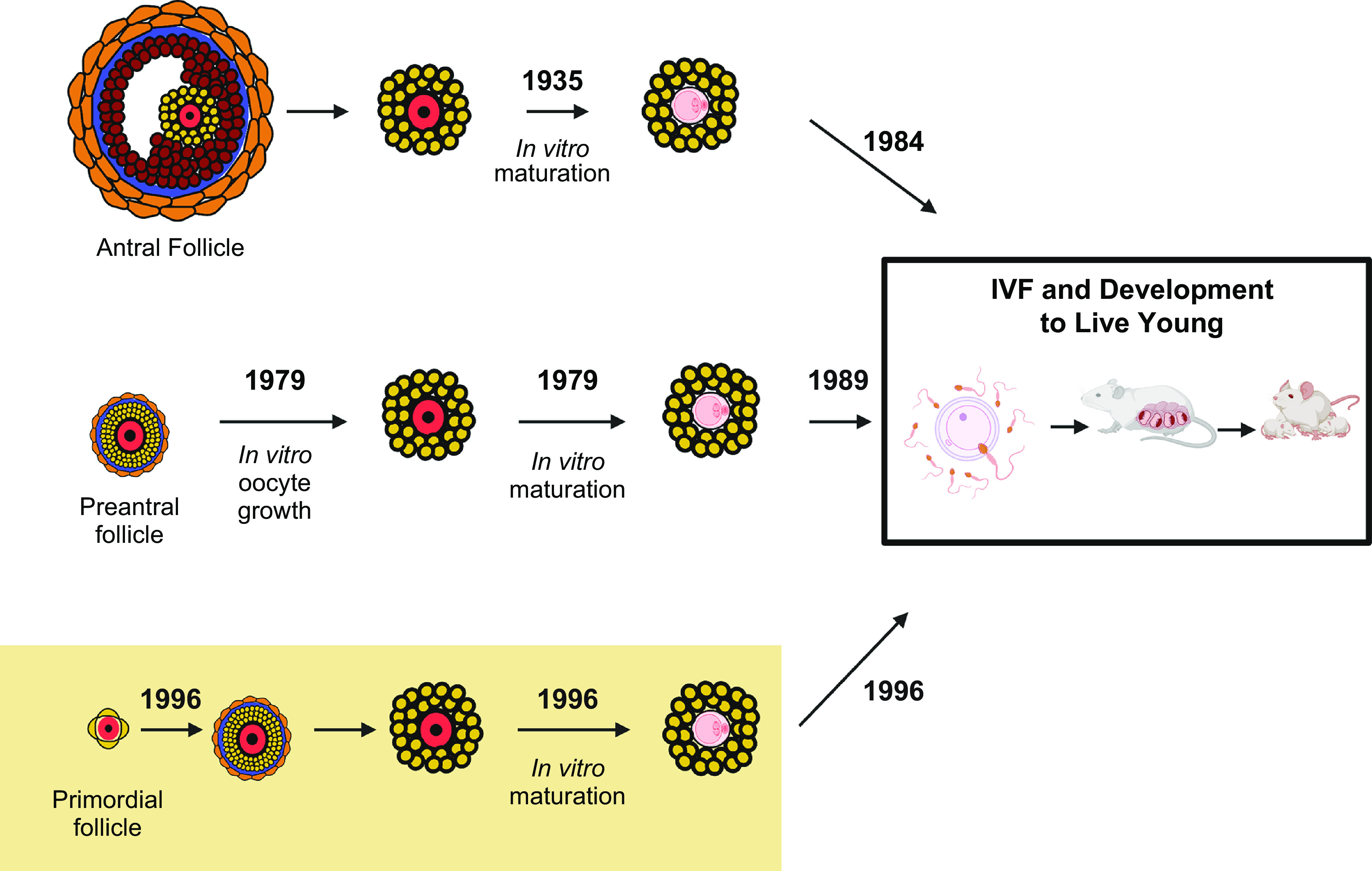
Development of mouse follicle/oocyte culture systems. Culture systems to support different stages of mouse oocytes through meiotic maturation, fertilization to the production of live young have been developed, initially starting with in vitro maturation of oocytes from antral stages (531), leading to successful fertilization and live young (532). Progressively moving to in vitro growth (IVG) of preantral follicles (533, 534) and now IVG of primordial follicles (535). IVF, in vitro fertilization. Image created with BioRender.com, with permission.

As far back as 1934 Pincus and Enzman (531) posed the question of whether mammalian oocytes could undergo normal development in vitro. Their work demonstrated that oocytes released from antral follicles spontaneously underwent meiotic maturation, marking the emergence of systems to support oocyte development in vitro. It took 50 years of research before in vitro matured mouse oocytes could be successfully fertilized and produce embryos and live young (532) (FIGURE 11). Systems to support mouse oocyte growth from preantral stages in vitro were developed over 40 years ago, and oocytes that could undergo maturation after a period of in vitro growth (IVG) were produced (533). Improvements in these systems led to the production of embryos and live young from oocytes derived from in vitro-grown preantral follicles (534) (FIGURE 11). This early work on mice provided models that advanced our knowledge of the regulation of oocyte development particularly relating to the role of oocyte-somatic cell interactions and the role of oocyte-secreted factors (536, 537).

Improved knowledge of factors regulating oocyte development led to advances in culture systems being made (538). It was subsequently demonstrated that the most immature stage of the follicle, primordial follicles, could be activated to grow in vitro, reach the preantral stage, then following a further period of culture, oocytes could be matured and fertilized in vitro (535, 539). These in vitro-grown oocytes produced viable embryos and live offspring (535, 539) (FIGURE 11). The in vitro development of primordial follicles required a two-step culture system and the initial work resulted in the birth of a single mouse that subsequently developed several abnormalities in adulthood (535). Following alterations in the culture medium, several mouse embryos and offspring were produced using oocytes that were in vitro grown (IVG), followed by IVM and IVF (539). This work provided proof of concept that complete oocyte development can be achieved in vitro and has driven the development of culture systems that could be applied to other species, particularly humans (reviewed in Refs. 540–542). Transferring these techniques from rodents to humans and large mammals has been challenging, in part due to the differences in the size of follicles and protracted duration of growth. Nonetheless, the advances in culturing follicles from humans, nonhuman primates, and domestic species that have been made, bring the prospect of human oocyte development in vitro, closer (7, 541).

### 13.2. Human Primordial Follicle Culture

**FIGURE 12.**
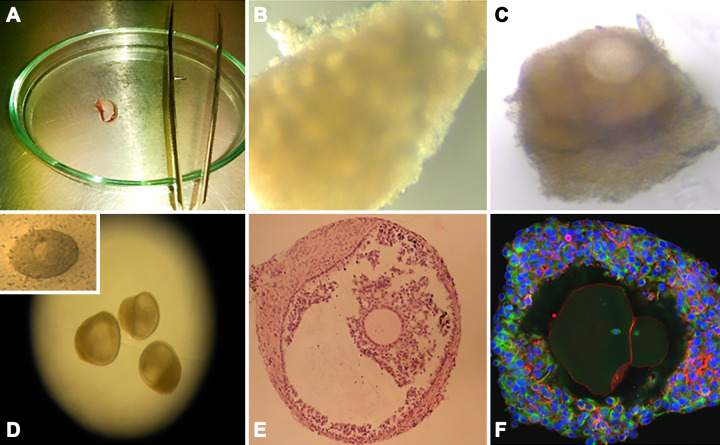
In vitro growth (IVG) of human primordial follicles. *A*: human ovarian cortical tissue piece prior to being prepared into microcortex for step one of culture. *B*: microcortex following *step 1* of culture, showing evidence of follicle activation. *C*: growing follicle with surrounding theca cells dissected from microcortex following 8 days of culture and selected for individual culture in *step 2* of multistep culture system. *D*: antral follicle formed following 8 days in *step 1* and a further 8 days in *step 2* of culture. *Inset*: removal of oocyte-granulosa cell complex for culture in *step 3*. *E*: histological section of in vitro grown antral follicle. *F*: confocal image of IVG and matured oocyte, characterized by the emission of a first polar body and meiotic spindle (543).

Supporting this complex multilayered process in vitro is technically challenging; however, complete human oocyte development from primordial/unilaminar follicles to meiotic maturation (i.e., at metaphase II) has been achieved (543, 544). Culture systems developed to support human oocyte development utilize either biopsies of ovarian cortex from healthy women or biopsies of whole ovaries that have been removed for fertility preservation (7). If the ovarian cortex is being used, then this tissue will contain mainly primordial follicles while growing follicles can be isolated from whole ovaries (FIGURE 12*A*).

As discussed in detail earlier, PFA is a defining step for oocyte development. During this process, primordial follicles respond to a combination of inhibitory and stimulatory factors, within the context of complex intrafollicular networks that integrate signals from various signaling pathways (182) (FIGURE 4). Early attempts at culturing isolated human primordial follicles could not activate their growth (545–547). It has now become clear that primordial follicles interact with surrounding stromal cells (153) and need to be maintained within small pieces of that stromal tissue. By maintaining the structural integrity of cortical tissue and preserving interactions between follicles and surrounding interstitial tissue, the survival and growth of primordial follicles can be maintained (376, 548–550). That primordial and early growing follicles do not have an intimate vascular supply is advantageous for maintaining fragments of ovarian cortex in vitro.

A spontaneous shift from quiescent primordial to growing follicles occurs within the cultured ovarian cortex over 6–8 days in vitro (376, 543) (FIGURE 12*B*). Extensive primordial follicle activation in vitro has been reported in nonhuman primates (551), bovine (552), caprine (553), and ovine (554). Activation of primordial follicles occurs gradually in a regulated manner in vivo and the accelerated activation observed in vitro is likely the consequence of disruption of inhibitory mechanisms that maintain primordial follicles in a quiescent state (237, 555). Given that the regulation of follicle growth is affected by tissue pressure and surrounding stromal cells (556) (vide supra), the manner in which the architecture of the ovarian cortex is prepared for culture will affect these biomechanical forces (557) and thus the degree of follicle activation. Human ovarian tissue being prepared for culture can be cut into fragments (micro cortex) with the removal of the growing follicles and the underlying stroma before mechanically loosening the fragments (376, 543) (FIGURE 12, *A* AND *B*). This microcortex preparation results in higher rates of activation (376, 543) compared to ovarian cortex cultured as dense cubes (547–549), further emphasizing the significance of biomechanical forces within the ovary in maintaining the balance between dormancy and activation of resting follicles (320, 558).

Tissue preparation and ovarian cortex remodeling have been linked to a disruption of the Hippo signaling pathway. Fragmentation of the ovarian cortex leads to an imbalance in the G-actin/F-actin ratio of the Hippo cascade, which alters the balance of negative regulators of growth leading to the translocation of Hippo’s main effector YAP into the nucleus of granulosa cells. Nuclear YAP allows the transcription of growth factors and apoptosis inhibitors, resulting in follicle awakening (215, 239).

This microcortex that has been fragmented and mechanically loosened supports activation of human primordial follicles that develop to multilaminar (secondary) stages within 6 days (376, 543) (FIGURE 12, *B* AND *C*). Secondary-stage follicles do not survive beyond this stage if they remain within the ovarian fragments, probably as a consequence of follicle interactions (283, 284) although the relevant signaling factors have yet to be identified; therefore, to avoid degeneration, they need to be isolated and grown individually (FIGURE 12*C*).

### 13.3. Preantral Follicle Culture

Multilaminar follicles can be isolated from ovarian stroma using enzymes such as collagenase and DNAse (559, 560) or Liberase (561, 562). However, the use of enzymes may damage the surrounding theca cell layers that are needed to retain follicular structure as well as being the source of factors supporting follicle growth and function (376, 563). Isolation of follicles by mechanical means (i.e., using needles) and without enzymes maintains the basal lamina and thecal layers, thus preserving follicle integrity (FIGURE 12*C*); however, it is a time-consuming process requiring skill and patience and results in a relatively low yield of follicles being isolated (213, 311, 376, 543).

Culture systems have been developed to support isolated multilaminar (preantral) growing follicles isolated from the human ovarian cortex (564–568) or developed in vitro from primordial stages (311, 376, 543, 544). Human ovarian follicles can grow up to several millimeters in diameter therefore, maintaining their structure in vitro is challenging and tissue-engineering principles have been applied to tackle this problem (540, 569). Several groups have encapsulated human preantral follicles within biomatrixes such as alginate to provide physical support (565, 567, 568). While alginate maintains follicle structure, the rigidity and pressure can negatively affect gene expression and follicle development (570). Alginate prepared at higher concentrations can inhibit the delivery of growth factors (571) and may impede ECM remodeling, interactions, and cell adhesion (572) unless it is combined with bioactive molecules (573).

Novel scaffolds to support human follicle growth in vitro have been developed; these include decellularized ovarian tissue (566, 574, 575) and three-dimensional microporous scaffolds (576, 577). The use of these scaffolds may enhance in vitro development of follicles but will be important in developing artificial ovaries for transplantation (569, 578).

**FIGURE 13.**
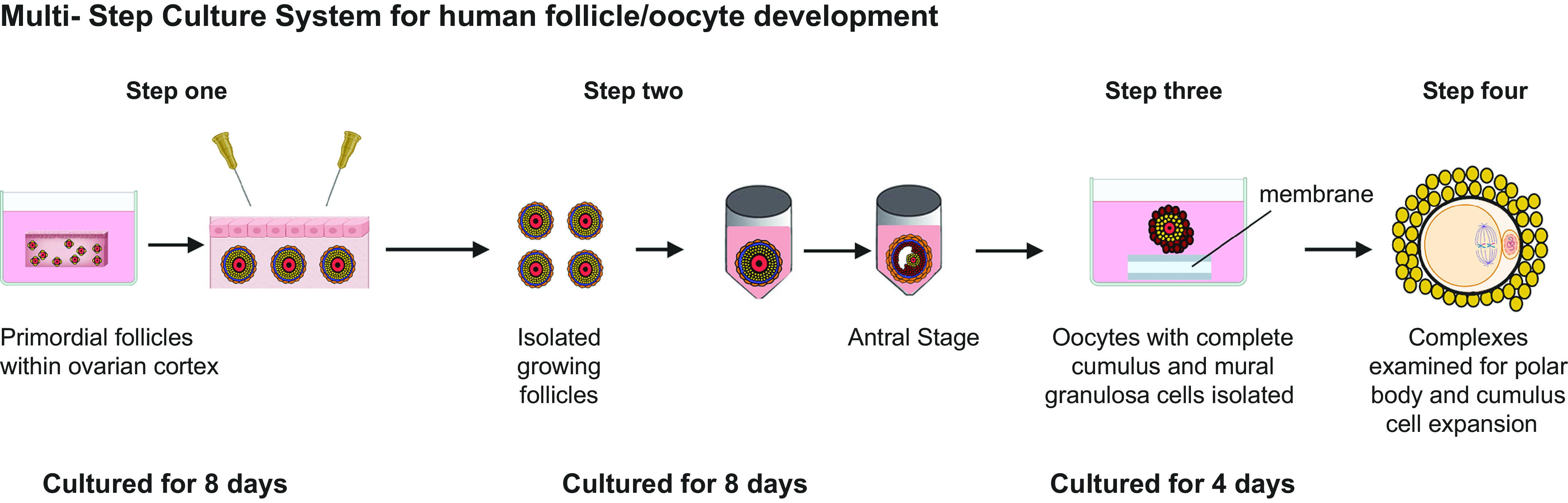
Multistep culture system for human oocyte development from primordial to maturity (376, 543). *Step 1*: pieces of ovarian tissue containing primordial/unilaminar follicles are prepared for culture. Once follicles have reached multilaminar stages, they can be mechanically isolated using needles. *Step 2*: isolated follicles are cultured individually from preantral to antral stages. *Step 3*: cumulus-oocyte complexes (COCs) are retrieved from the antral follicles and further cultured until oocyte diameter is >100 µm. *Step 4*: COCs are placed within medium for in vitro maturation (IVM) and then examined for cumulus cell expansion (yellow), Metaphase II spindle formation and the presence of a polar body (543). Image created with BioRender.com, with permission.

Importantly, a supporting matrix is not required to promote the development of isolated human follicles when the theca layer is intact, and this may also support more physiological interactions between the compartments of the follicle (543) (FIGURE 12, *A*–*F*, AND FIGURE 13). In this multistep culture system, isolated multilaminar follicles that were placed individually within v-shaped microwell plates with serum-free medium containing a low concentration of FSH, activin A, [previously shown to support follicular integrity in cultured bovine follicles (378)], and ascorbic acid [that reduces cell death in cultured preantral follicles (579)] maintain their three-dimensional structure (543) (FIGURE 13). Under these conditions, human follicles grow and form antral cavities with differentiated granulosa cells (213, 311, 376, 543) and antral cavities forming within 10 days (FIGURE 12, *D* AND *E*). Oocyte-granulosa cell complexes (OGCs) can then be removed from the antral follicles by applying gentle pressure to the follicle (543). Intact OGCs with complete cumulus and adherent mural granulosa cells are selected for further growth on membranes in *step 3* of the multistep system (FIGURE 12*D*).

The culture system that supports the growth of mouse oocytes from primordial stages focuses on OGCs to stimulate oocyte development rather than growing intact antral follicles to preovulatory stages in vitro (539). The reasoning for developing *step 3* (i.e., isolated OGCs) of the human multistep culture system is informed by this mouse system as culturing human preantral follicles to in vivo preovulatory sizes (20 mm or more in diameter in vivo) is technically challenging. Thus, the aim of *step 3* is to promote oocyte growth as oocyte size is an indicator of meiotic and developmental potential (580). Following this step, oocytes of at least 100 μm in diameter can be obtained and selected for IVM to resume meiosis and reach metaphase II (543) (FIGURE 12*F*).

Sustaining the oocyte-somatic cell interactions that are essential to support oocyte development is fundamental for a successful culture system. Regulation of cell-cell communication is controlled by members of the TGF-β superfamily, including GDF-9 and BMP-15 (317). In the human ovary, these are expressed in both the oocyte and cumulus granulosa cells where they support the establishment and maintenance of cell-cell interactions as well as stimulating cell proliferation (581) (described in more detail earlier). Improved oocyte maturation and fertilization rates in humans (582) have been linked to increased mRNA levels of both GDF-9 and BMP-15 in cumulus cells and the addition of either factor to cultured human ovarian cortex has resulted in increased PFA (583).

### 13.4. Final Meiotic Maturation (IVM)

At the end of the IVG process, isolated complexes must undergo IVM to support the resumption of meiosis to the point of completing metaphase I and reaching metaphase II. IVM of immature human oocytes has been developed for over 50 years (584) but it took until 1991 before the first live birth following IVM was reported (585) reviewed in (586). Maturation rates of immature oocytes are less than that of oocytes collected from stimulated ovaries (587) and while this reflects the quality of oocytes harvested for IVM it also emphasizes the need for improvements in IVM protocols (588).

A significant advance in IVM that involves a prematuration phase has been made recently (589, 590). This technique inhibits spontaneous meiotic maturation that occurs in vitro while maintaining synchronization of oocyte (nuclear and cytoplasmic) maturation. The prematuration step is carried out in the presence of C-type natriuretic peptide [“capacitation” step (CAPA)], followed by conventional IVM (overall termed CAPA-IVM). There is now accumulating evidence that CAPA-IVM increases oocyte maturation rates and leads to enhanced embryo quality and higher pregnancy rates than conventional IVM alone (591–593). Remarkably, a recent RCT has shown noninferiority of first-cycle live birth rate with CAPA-IVM compared to conventional IVF in women at high risk of ovarian hyperstimulation (594) although the cumulative ongoing pregnancy rates at 12 months after randomization were 44.0% in the IVM group and 62.6% in the IVF group (absolute risk difference: −18.7%; 95% confidence interval: −27.3%, −10.1%). Thus, further refinement and optimization of IVM protocols are necessary to develop and validate a standardized, efficient, and safe IVM system and enhance maturation rates and developmental potential of IVG-derived oocytes, but the potential advantages in simplification and cost-reduction by avoiding ovarian stimulation are considerable.

A proportion of IVG oocytes resulting from the multistep culture system described above can undergo meiotic maturation following IVM. Approximately 30% of oocytes that survive the entire culture period go on to form metaphase II spindles (543) (FIGURE 12*F*). The polar bodies formed by these IVG oocytes are much larger than normal (543). The cause of these large polar bodies is as yet unknown, but the proximity of the spindle to the oocyte cortex is known to impact polar body size (595), and if contact between the spindle and the oocyte cortex is altered, this can result in the extrusion of large polar bodies (596). More recent work utilizing a multistep culture system over a prolonged period (9 weeks) has resulted in the successful maturation of IVG oocytes to the metaphase II stage following IVM with normal-sized polar bodies (544). It remains to be determined whether these IVG mature oocytes are developmentally competent. Further studies are needed to compare IVG mature oocytes derived from culture systems that differ in overall culture period, i.e., fast systems (543) or slower systems (544) to determine how oocyte function, chromosome arrangement, epigenetic imprinting, and health are impacted by culture period. The capacity to follow human oocyte development from primordial stages to maturation in vitro provides insight into the basic science of oogenesis, folliculogenesis, and meiosis and could lead to the development and improvement of ARTs.

As well as developing oocytes in vitro, progress has been made in deriving oocytes from stem cells entirely in vitro. The in vitro derivation of oocytes provides important models for research, but if oocytes derived in this way are shown to be developmentally normal, they could be used clinically. An in vitro-derived source of oocytes would reduce the need for donor eggs as well as increase fertility preservation/restoration options for a range of patients.

## 14. IN VITRO DERIVATION OF OOCYTES

In vitro systems have been developed to derive oocytes from mouse pluripotent stem cells (PSCs) (597), and these have greatly enhanced our knowledge of the processes of germline differentiation and development. The development of human models is now highlighting divergence and key differences in the molecular identity of human and mouse germ cell development (530). The in vitro derivation (IVD) of oocytes from stem cells has a clear application in furthering our understanding of oocyte development, and while there have been rapid advances using mouse models, progress is also being made utilizing human PSCs.

Pluripotent cells that have been utilized experimentally are embryonic stem cells (ESCs) or induced Pluripotent stem cells (iPSCs). Two types of stem cell lines have been derived from the inner cell mass of the developing blastocyst that forms the pluripotent epiblast cells: *1*) ESCs (598), and *2*) epiblast stem cells (EpiSCs) (599). These cell lines can differentiate into somatic and germline lineages (599). ESCs have been derived from human blastocysts (hESCs) (600) and are candidate progenitor cells for in vitro oogenesis (601).

While research with mouse ESCs (mESCs) gives us insight into cell lineage development and proof of principle, the use of human ESCs clinically is fraught with practical difficulties and ethical concerns. There are ethical concerns surrounding the use of human embryos and stem-based research in general (602). Concern in relation to the derivation of oocytes from human ESCs for clinical application is that somatic cell nuclear transfer would be required to make the cells biologically related to the recipient (603). Given the myriad of concerns, it is unlikely that derivation of gametes by this route would be applied clinically, and a more likely route would be to utilize iPSCs derived from a woman’s own adult cells, which overcomes the difficulties associated with hESCs and nuclear transfer (601).

Methodology to dedifferentiate and induce pluripotency in adult cells was developed by Takahashi and Yamanaka (604, 605) when mouse fibroblasts, under the expression of four crucial pluripotency genes, Oct3/4, Klf4, Sox2, and c-Myc, were induced to a pluripotent state and termed iPSCs. Soon after, iPSCs were derived from several species including humans (604). These cells have been used to regenerate several tissue types and are becoming clinically viable (606). Given that iPSCs do not raise the same ethical concerns as ESCs, they are more likely to provide a clinical option for deriving oocytes.

### 14.1. Functional Oocytes from Stem Cells

Differentiating germ cells have been derived from mouse embryonic stem cells (mESCs) (607). Epiblast-like cells (EpiLCs) has been induced from mESCs with epigenetic changes replicating in vivo differentiation of epiblast cells into primordial germ cells (608). The successful differentiation of EpiLCs to primordial germ cell-like cells (PGCLCs) in vitro was analogous to that occurring in vivo. PGCLCs derived from embryoid bodies (EB) have differentiated into oocyte-like cells and expressed early meiotic markers and oocyte-specific genes (FIGURE l*A*, *gdf9*, *zp1*, *zp2*, and zp3) when cocultured with granulosa cells (609), with similar results observed coculturing PGCLCs with Chinese hamster ovary cells (610). However, these results could not be replicated with granulosa cell conditioned medium (609), confirming the importance of cell-cell interactions with ovarian somatic cells.

Follicle-like structures containing oocyte-like cells (OLCs) have been formed by combining PGCLCs with embryonic ovarian somatic cells following transplantation to the ovarian bursa of immune-deficient recipient mice. The OLCs were capable of being matured and fertilized in vitro and embryos were produced, albeit with lower efficiency than with normal oocytes, resulting in healthy offspring with normal imprinting patterns (611). These studies demonstrated the potential of mESCs to undergo differentiation to all stages of oogenesis and subsequent embryonic development.

The ability to generate all cell types including germ cells from mouse iPSCs has been demonstrated to be similar to ESCs (612, 613). Mouse iPSCs have subsequently been derived to EpiLCs and PGCLCs in vitro and have formed a reconstituted ovary after being combined with fetal ovarian somatic cells, and following xenotransplantation oocytes have been generated (reviewed by Ref. 597). Competent oocytes can now be derived from stem cells entirely in vitro, avoiding the need for a transplantation stage (614). Hikabe et al. (614) developed a multistep system that supports in vitro differentiation, in vitro growth, and in vitro maturation to produce developmentally competent oocytes entirely in vitro. Some of the oocytes reached metaphase II and were fertilized; however, less than 4% of oocytes resulted in the formation of embryos, but healthy offspring were produced.

**FIGURE 14.**
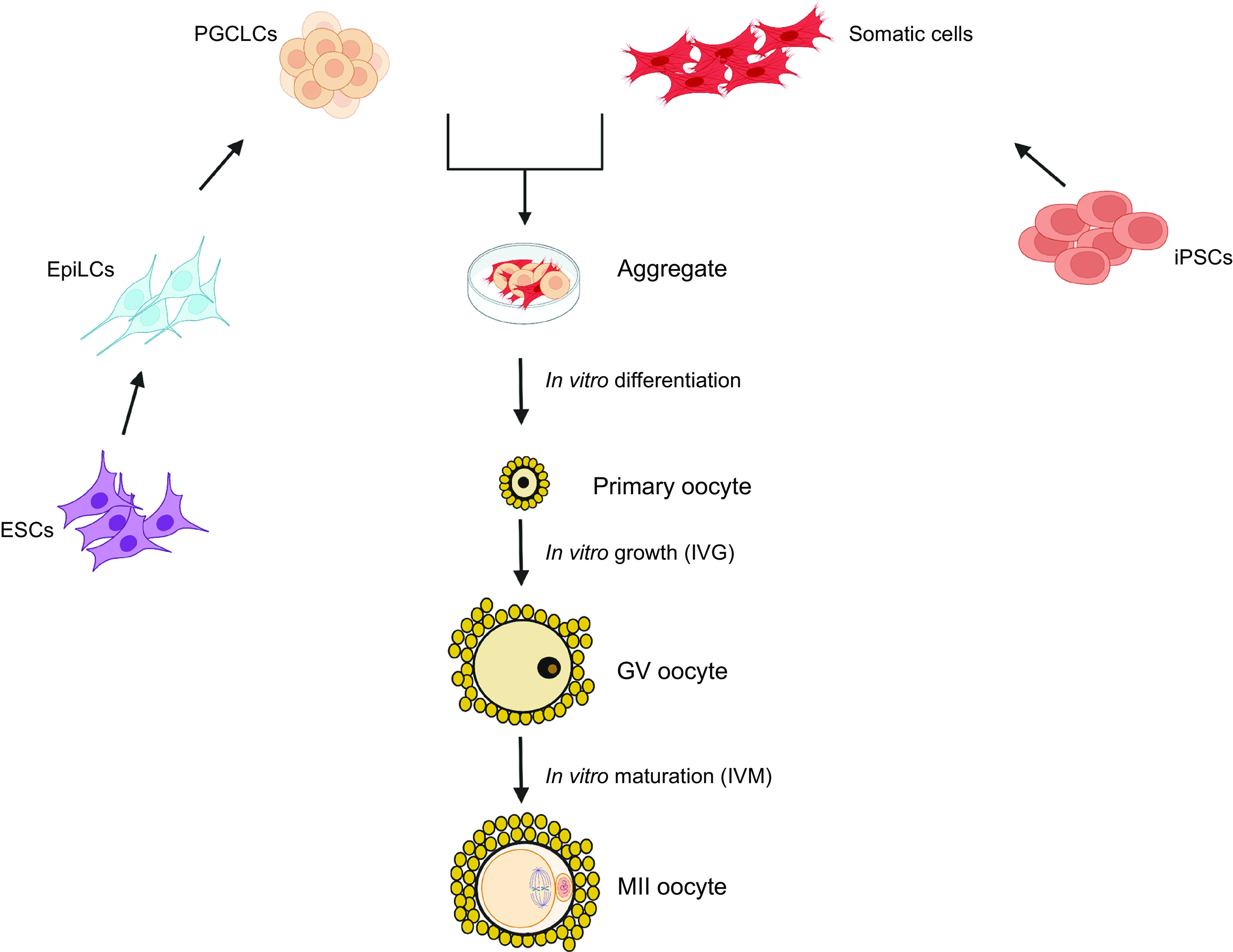
In vitro derivation of mouse oocytes from stem cells. Complete in vitro formation of ovarian follicles from embryonic stem cells (ESCs) or induced pluripotent stem cells (iPSCs) (613, 614) used embryonic tissue to obtain somatic cells to support germ cell development, whereas (614, 615) derived somatic support cells from iPSCS demonstrated the complete recapitulation of germ cell development in vitro forming competent oocytes capable of being fertilized and forming embryos. MII, metaphase II; PGCLCs, primordial germ cell-like cells; EpiLCs, epiblast-like cells. Image created with BioRender.com, with permission.

The ability to derive oocytes from stem cells entirely in vitro is a huge step, but this protocol (614) relied on using embryonic tissue as a source of somatic cells to support germ cell development. The use of embryonic/fetal tissue is not a viable option if these protocols are ever to be applied to humans and utilized clinically. However, this hurdle now appears to have been surmounted with the development of the necessary ovarian somatic cell support from stem cells (615) (FIGURE 14). Under defined culture conditions, mESCs can be differentiated into fetal ovarian somatic cell-like cells (FOSLCs) (615) and these can be combined with PGCLCs also derived from mESCs to form aggregates that support the formation of follicles, with functional oocytes, which are capable of being fertilized, forming embryos that then develop into healthy offspring (615). The ability to form functional oocytes/follicles without the need to utilize embryonic somatic cells (although thus far still using embryonic stem cells) is a major advance and sets the scene for developing support cells from iPSCs derived from adult cells, thus moving considerably closer to useful techniques for humans and other species (597) (FIGURE 14). The necessity for sex-specific germ cell development, demonstrated by many studies showing that sex-reversed XY or XX germ cells have poor developmental competence, has been challenged by recent work providing proof of concept that functional oocytes can be produced from stem cells derived from male mice (616). This involved selection for the spontaneous loss of the Y chromosome and duplication of the X, thus converting the XY chromosome set to XX. The complexities of these manipulations mean that clinical application is distant.

### 14.2. Oocytes from Human Stem Cells

Germ cell differentiation from human ESCs has been investigated, and PGCLCs have been derived from hESCs with gene expression patterns similar to PGCs (617). The differentiation of hESCs to germ cell precursors occurs spontaneously but the addition of growth factors such as BMP4 increases the rate of differentiation (618). Several growth factors and feeder layers have been used to improve differentiation and cells with germ line and meiotic markers have been obtained (reviewed in Ref, 619). More recently, hESCs have developed into oocyte-like structures (620), but meiosis was not observed. PGCLCs have been induced from human iPSCs (hiPSCs) (621, 622), and postmeiotic germ-like cells have been formed (623). PGCLCs and oogonia derived from hiPSCs and combined with human fetal-derived somatic cells formed follicle-like structures (624), and recently, advances have been made in forming human granulosa-like cells from hiPSCs (625). These developments bring us closer to human oocytes/follicles being derived entirely in vitro from hiPSCs and represent major progress in defining the mechanisms required to produce functional and high-quality oocytes (626) as well as moving considerably closer to producing “artificial gametes” for clinical use.

## 15. SUMMARY

This review has highlighted the complexity of human oocyte development within the context of the ovarian follicle and the dynamic ovarian environment and its alterations through aging. The control of these processes is multilayered involving paracrine, autocrine, and endocrine regulators, as well as new layers of complexity such as through small noncoding miRNAs that modify gene expression. New factors and pathways continue to be identified through single-cell sequencing and bioinformatics. Our understanding of these processes has been informed by mouse models and using large mammals, enhanced by human in vitro models, and these have facilitated the identification of targets that may have utility for therapeutic purposes. We are moving closer to developing therapies that will protect the follicle reserve from damaging chemotherapeutic agents and environmental insults to alleviate or prevent POI and even potentially reduce the adverse effects of aging on oocyte quality. Indeed, remarkable data have been very recently obtained using drugs that are already in clinical practice, making treatments for a range of ovarian conditions within immediate reach. As well as facilitating a greater understanding of the mechanisms regulating normal human oocyte development, the processes of IVD and IVG could ultimately contribute to new approaches to the treatment of infertility and, with further developments in IVM, become an integral part of fertility preservation for young girls and women. Before these methodologies can progress toward clinical application they need to be shown to be reliable and safe, but it is clear that such developments are approaching.

## GRANTS

The authors’ work in this field is supported by grants from the Wellcome Trust (215625/Z/19/Z to E.E.T. and R.A.A.) the Medical Research Council (MR/T025654/1 to E.E.T, MR/R003246/1 to E.E.T. and R.A.A, G1100357 and MR/W019140/1 to R.A.A, and MR/N022556/1 to the MRC Center for Reproductive Health), Wellbeing of Women (PRF005 to R.R.), and Biotechnology and Biological Sciences Research Council (BB/R015635/1 to R.A.A.).

## DISCLOSURES

No conflicts of interest, financial or otherwise, are declared by the authors.

## AUTHOR CONTRIBUTIONS

E.E.T., J.G., Y.L.O., and R.R. prepared figures; E.E.T. and R.A.A. drafted manuscript; E.E.T., J.G., Y.L.O., R.R., and R.A.A. edited and revised manuscript; E.E.T., J.G., Y.L.O., R.R., and R.A.A. approved final version of manuscript. 
